# Cascade Therapy of Periodontitis via Sequential Release of Ribosome‐Targeting Antimicrobial Peptide and Irisin From a Multifunctional MOF‐Based System

**DOI:** 10.1002/advs.202521553

**Published:** 2026-01-27

**Authors:** Yan Chen, Zheng Xu, Yunmo Xue, Shanshan Liu, Xiang Zhang, Jingyao Guo, Minhui Yao, Yue Liu, Xiaolin Lu, Jieshu Qian, Qian Ma

**Affiliations:** ^1^ Department of General Dentistry the Affiliated Stomatological Hospital of Nanjing Medical University State Key Laboratory Cultivation Base of Research, Prevention and Treatment for Oral Diseases Jiangsu Province Engineering Research Center of Stomatological Translational Medicine Nanjing Jiangsu China; ^2^ State Key Laboratory of Digital Medical Engineering School of Biological Science and Medical Engineering Southeast University Nanjing China; ^3^ Department of Stomatology the First Affiliated Hospital of Bengbu Medical University Bengbu China; ^4^ School of Environmental and Biological Engineering Nanjing University of Science and Technology Nanjing China; ^5^ Jiangsu Key Laboratory for Recognition and Remediation of Emerging Pollutants in Taihu Basin, School of Environmental Science and Engineering Wuxi University Jiangsu PR China

**Keywords:** periodontitis, metal‐organic frameworks, cascade therapy, antimicrobial peptides, irisin

## Abstract

Surgical removal of bacterial plaque and antibiotic therapy have been widely used for the clinical treatment of periodontitis, which is driven by microbial dysbiosis and usually causes the loss of alveolar bone. However, key restrictions including drug resistance, poor anti‐inflammatory effects, and limited bone repair capacity exist in antibiotic therapy, presenting challenges for periodontitis treatment. Consequently, it is urgently required to develop novel systems to combat antimicrobial resistance and enhance bone regeneration ability. Here, a MOF‐based multicomponent system is developed, containing a cationic antimicrobial peptide GF for antimicrobial purpose and the adipomyokine Irisin for anti‐inflammation, antioxidation, and promoting bone regeneration. The obtained composite Irisin/GF@NH_2_‐MIL‐101(Fe) exhibits pH‐responsive release of GF and irisin. In vitro and vivo experiments demonstrate this multicomponent system has robust antimicrobial activity and could attenuate inflammation while stimulating bone regeneration. Remarkably, the antimicrobial mechanism of GF is explored, revealing a distinct binding mode at the ribosomal A‐site, which establishes more stable interactions than tetracycline, thereby disrupting protein synthesis and effectively reducing the risk of antibiotic resistance. This study not only reveals a novel antibacterial mechanism of antimicrobial peptide, but also provides a novel cascade therapy for the treatment of periodontitis with multiple functions.

## Graphical abstract

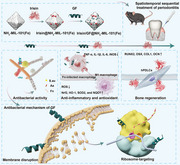

## 1 Introduction

Periodontitis is a chronic, multifactorial inflammatory disease associated with the disruption of dental plaque biofilms [1]. Its progression is driven by the overgrowth of periodontal pathogens, activation of the host's immune‐inflammatory response, and subsequent alveolar bone loss [2, 3, 4]. This self‐feeding cycle of infection, inflammation, and bone destruction results in tooth loss and contributes to systemic complications [5, 6]. Although antibiotics remain a common clinical strategy for managing periodontal infections, their effectiveness is increasingly challenged. Numerous studies have reported that key periodontal pathogens, e.g., *Fusobacterium nucleatum* (F. *nucleatum*), *Porphyromonas gingivalis* (P. *gingivalis*), and *Aggregatibacter actinomycetemcomitans* (A. *actinomycetemcomitans*), have developed resistance to commonly prescribed antibiotics including tetracycline, clindamycin, amoxicillin, and metronidazole [7, 8, 9]. Moreover, a single antibiotic often fails to eliminate the complex microbial communities within subgingival biofilms, thus limiting the therapeutic outcome [10]. While local or systemic antibiotic administration can temporarily reduce microbial load and alleviate symptoms, long‐term use normally brings risks such as microbial dysbiosis, allergic reactions, and systemic side effects [9, 11]. Moreover, conventional antibiotic therapies primarily target the elimination of pathogens but fall short in restoring periodontal homeostasis and promoting alveolar bone regeneration [12, 13]. Consequently, extensive research efforts have been made in developing stage‐adaptive controlled release systems that can coordinate the spatiotemporal sequential demands of antibacterial, anti‐inflammatory, and bone‐remodeling processes during periodontitis treatment.

**FIGURE 1:**
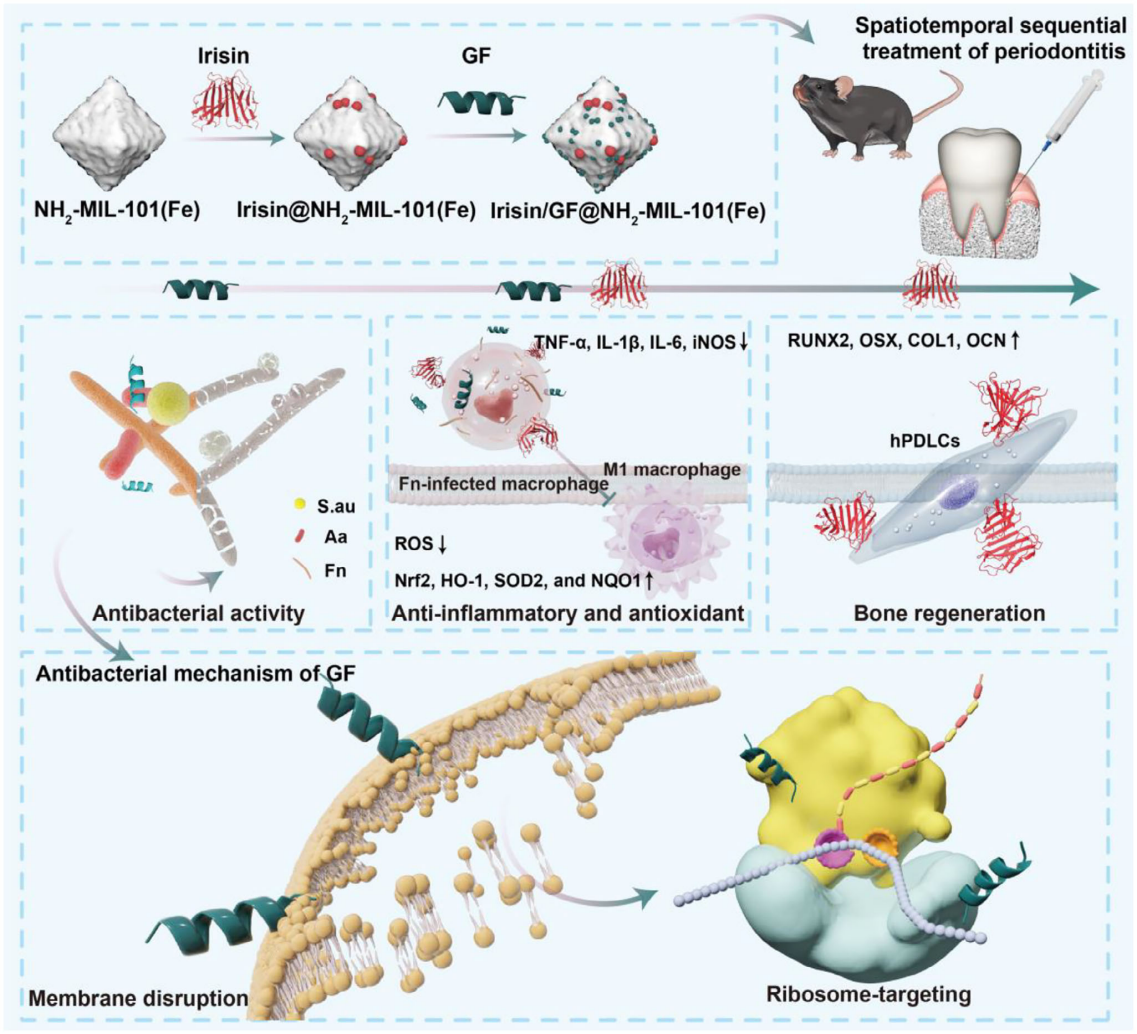
Schematic illustration of this study. This study presents a novel strategy for periodontitis treatment by co‐delivering a membrane‐ and ribosome‐targeting antimicrobial peptide GF and Irisin through a pH‐responsive metal‐organic framework. The system enables sequential release, providing rapid antibacterial action, anti‐inflammatory and antioxidative effects, as well as promoting bone regeneration, offering a promising solution to combat antimicrobial resistance and enhance tissue repair in periodontitis.

Antimicrobial peptides (AMPs) are broad‐spectrum antibacterial agents that act through multiple mechanisms, including disrupting microbial membranes and inhibiting protein or nucleic acid synthesis, thereby reducing the risk of antibiotics resistance [14, 15]. However, their clinical application is hindered by issues such as cytotoxicity, poor stability, and the time‐ and cost‐intensive development. These challenges have driven the development of rational design strategies that optimize AMP structures at the molecular level to improve structural stability, safety, and antibacterial efficacy [16, 17]. Differences in AMP sequence and conformation can also influence their antimicrobial behavior by affecting membrane interactions or intracellular activities [18]. AMPs enriched in positively charged amino acids, such as lysine and arginine, exhibit a cationic nature that enables electrostatic interactions with negatively charged phospholipids on bacterial membranes, while the distribution of hydrophobic residues facilitates membrane insertion and disruption [19, 20]. Distinct secondary structures, including α‐helices and β‐sheets, further modulate the modes of interaction between antimicrobial peptides and bacterial membranes or intracellular targets. In particular, α‐helical peptides often undergo conformational transitions in membrane environments to enhance membrane binding, whereas β‐sheet antimicrobial peptides exert their antibacterial effects through stable conformations or by targeting intracellular sites [21, 22]. In a recent study, we designed and optimized a highly effective AMP derivative, GF, with GLFKIIKKIAKSF peptide sequence, from natural peptide sequences using sum frequency generation (SFG) spectroscopy and molecular dynamics (MD) simulations. Previous findings demonstrated that introducing a terminal phenyl group markedly strengthens the peptide's membrane interfacial interactions, thereby enhancing its capacity to insert into and disrupt bacterial membranes [23]. Consistent with these mechanistic observations, GF exhibited potent antibacterial activity against key oral pathogens, including A. *actinomycetemcomitans*, *Streptococcus mutans* (S. *mutans*), and *Staphylococcus aureus* (S. *aureus*), with minimum inhibitory concentrations (MICs) below 7.5 µg/mL. Compared with natural AMPs such as hBD3 and LL‐37, these values are reported to be lower [23, 24, 25]. In addition, GF has also possessed enhanced antibacterial efficacy against methicillin‐resistant Staphylococcus aureus (MRSA) than commercially available antibiotics, suggesting its promising potential for combating multidrug‐resistant bacteria. Furthermore, GF has a relatively small molecular weight (<1200 Da), which facilitates its penetration through bacterial membranes and supports cost‐effective and scalable synthesis [26, 27]. These features facilitate GF as a strong candidate to replace conventional antibiotics in periodontitis treatment. Although GF exhibits powerful membrane‐disruptive activity, its intracellular antibacterial mechanism remains unclear which limits its broader application within the oral field. This study investigates GF's intracellular actions and identifies potential molecular targets, thereby providing a theoretical foundation for future design of GF‐based periodontitis treatment agents.

In addition to antimicrobial therapy, effective treatment of periodontitis also requires the management of inflammation and enhancement of alveolar bone regeneration. Irisin, derived from the cleavage of fibronectin type III domain‐containing protein 5 (FNDC5) [28, 29], has been found to be expressed in human periodontal ligament cells (hPDLCs) and enhance their osteogenic differentiation [30, 31]. Given the immunological complexity of periodontitis, immunomodulatory strategies that target dysregulated inflammatory responses offer promising therapeutic avenues [32]. Recent studies have revealed that Irisin modulates macrophage activity and mitigates oxidative stress by regulating key signaling pathways such as SIRT3 and p38 MAPK, contributing to the restoration of molecular balance within the inflamed periodontal microenvironment [33, 34, 35]. These findings imply that Irisin could be used as a highly promising biologic agent for the immunomodulatory treatment of periodontitis.

Despite their promising therapeutic potential, the clinical application of peptide‐ and protein‐based therapeutics is often hindered by their short half‐life and susceptibility to enzymatic degradation [36]. Therefore, it is of great necessity to preserve the bioactivity of both GF and Irisin and achieve a responsive, sequential release to address the dynamic and complex tissue repair needs at different stages of periodontitis.

Various types of materials have been explored for controlled drug delivery [37]. However, organic carriers often suffer from limited loading capacity and insufficient stability, whereas inorganic materials may pose concerns regarding long‐term biocompatibility [38, 39, 40]. Metal–organic frameworks (MOFs), composed of metal ions or clusters coordinated with organic ligands into highly porous 2‐ or 3D networks, have drawn increasing attention in this context [41]. They can be tailored in terms of surface functional groups, pore environments, and morphology, enabling the stabilization of sensitive biomolecules under harsh conditions [42]. Moreover, their versatile chemical modifiability allows MOFs to be engineered as stimuli‐responsive platforms for controlled drug release [43].

In this study, we selected NH_2_‐MIL‐101(Fe), a representative MOF, as the delivery platform for the AMP and the adipomyokine. Among various MOF families, MIL‐type structures offer several advantages, including large surface area, tunable pore structure, excellent biocompatibility, and pH‐responsiveness, which make them well‐suited for drug delivery applications [44, 45]. Specifically, the iron ions (Fe^3+^) in NH_2_‐MIL‐101(Fe) act as strong Lewis acids, which not only provide optimal coordination with protein surfaces to facilitate efficient incorporation, but also enhance the structural stability of the MOF [46, 47, 48]. Moreover, the amino functional groups on the surface could enhance peptide loading through electrostatic interactions [49]. These properties together enable efficient encapsulation and sustained release of both GF and Irisin.

This study focuses on the antimicrobial peptide GF, exploring its intracellular mechanism through prokaryotic transcriptome sequencing and MD simulations. Results revealed that, in addition to its known membrane‐disrupting mechanism, GF also interferes with ribosomal transcription and translation. Furthermore, GF adopts a binding mode at the ribosome distinct from that of tetracycline, which may greatly reduce the risk of antibiotic resistance. These results inspired us to develop a multicomponent delivery system using NH_2_‐MIL‐101(Fe) as a carrier to support GF and the adipomyokine Irisin, namely Irisin/GF@NH_2_‐MIL‐101(Fe). Material characterization and both in vitro and in vivo experiments demonstrated that this system enables the pH‐responsive and rapid release of GF to control early‐stage infection, followed by the subsequent release of Irisin. This sequential delivery strategy achieves cascade therapeutic effects including antimicrobial, anti‐inflammatory, antioxidant, and osteogenic functions, which hopefully stimulate more studies toward the precision treatment of periodontitis (Figure 1).

## 2 Results and Discussion

### 2.1 GF Targets the Ribosome of F. *nucleatum* Beyond Membrane Disruption

F. *nucleatum* is one of the most prevalent anaerobic bacteria within the oral environment and is commonly observed in both healthy and diseased individuals. It is one of the dominant species within the oral microbial community. Numerous studies have established a close association between F. *nucleatum* and various forms of periodontitis and peri‐implantitis [50, 51, 52, 53]. F. *nucleatum* expresses multiple adhesins and serves as a key bridging factor in dental plaque biofilm formation by facilitating the adherence and aggregation of other pathogenic bacteria [54, 55]. In addition, F. *nucleatum* exhibits strong invasive capabilities, able to penetrate and enter various host cell types, with a higher degree of invasiveness compared to P. *gingivalis* [56, 57]. Moreover, F. *nucleatum* plays a role in the development of periodontal disease by triggering inflammatory responses through activation of the host immune system, leading to dysregulation of cytokine expression and subsequent tissue destruction. Given its critical role in biofilm development, host cell invasion, and inflammation, F. *nucleatum* was chosen as the model pathogen here to comprehensively evaluate the antimicrobial potential of GF for periodontal therapy.

The antimicrobial peptide GF which was designed based on a previously reported sequence (GLFKIIKKIAKSF) and structure has been found to exert bactericidal effects by disrupting bacterial membranes. GF demonstrated broad‐spectrum antibacterial activity at relatively low concentrations (≤10 µm) and effectively inhibited key oral pathogens associated with periodontitis and dental caries, suggesting its potential for managing polymicrobial infections in the oral cavity [23].

However, beyond its known membrane‐disruptive mechanism, GF may also act through additional intracellular targets. Notably, the mechanisms by which it affects Gram‐negative anaerobic bacteria remain largely unexplored. Within this study, we further investigated the antibacterial activity of GF against F. *nucleatum* and explored whether GF inhibits intracellular targets, aiming to uncover novel antimicrobial mechanisms beyond membrane disruption.

**FIGURE 2:**
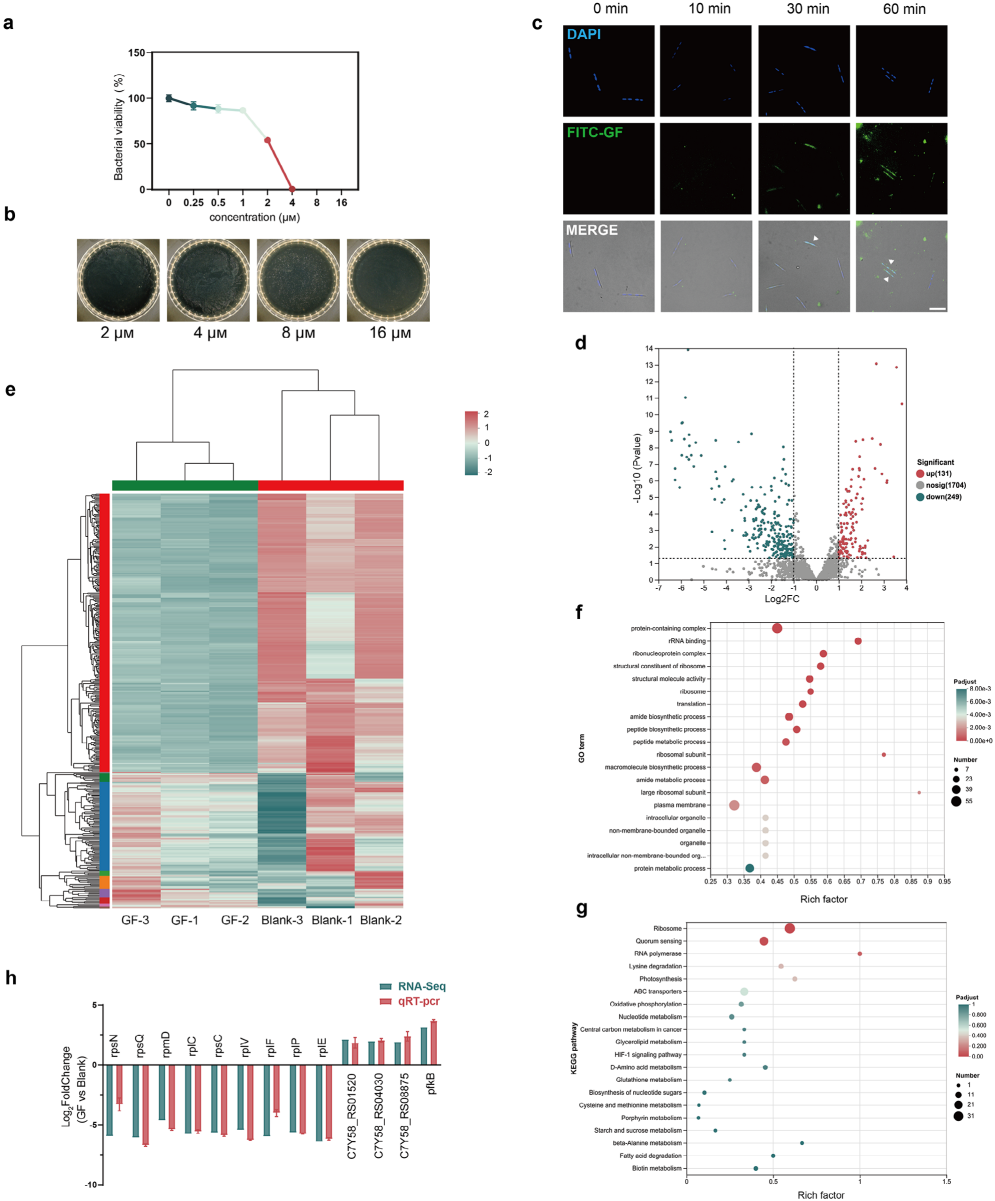
Antibacterial mechanism and transcriptomic analysis of GF. (a) Bacterial viability of F. *nucleatum* after 24 h treatment with different concentrations of GF (0–16 µm). (b) Corresponding agar plate assay results. (c) Fluorescence microscopy images of F. *nucleatum* stained with DAPI to label nucleic acids and co‐incubated with FITC‐GF for 0, 10, 30, and 60 min; white arrows indicate F. *nucleatum* with internalized GF (scale bar: 10 µm). (d) Volcano plot of DEGs between GF‐treated and control groups. (e) Cluster heatmap of DEGs. (f) GO enrichment analysis of DEGs. (g) KEGG pathway enrichment analysis of DEGs. (h) qPCR validation of key DEGs. Data in (a) and (h) are presented as means ± SD (n = 3).

We first assessed the antibacterial activity of GF against F. *nucleatum*. As shown in Figure 2a,b, GF exhibited a MIC of 4 µg/mL and a minimum bactericidal concentration (MBC) below 16 µg/mL, indicating strong bactericidal efficacy against F. *nucleatum*. To investigate whether GF targets intracellular sites, we labelled GF with Fluorescein isothiocyanate (FITC) and monitored its interaction with F. *nucleatum* in real time. Microscopy results (Figure 2c) showed that GF was internalized by F. *nucleatum* within 30 min of incubation and remained inside the cells for up to 60 min, suggesting that GF may exert antibacterial effects through intracellular mechanisms in addition to membrane disruption.

To further investigate the intracellular effects of GF, we conducted a comparative transcriptomic analysis between F. *nucleatum* treated with GF and untreated controls. Principal component analysis (PCA) (Figure S1) exhibited a distinct separation between the two groups along the PC1 axis, indicating that GF treatment caused substantial changes in the bacterial gene expression profile. Visualization using a volcano plot (Figure 2d) revealed 131 upregulated and 249 downregulated differentially expressed genes (DEGs). Heatmap analysis (Figure 2e) further demonstrated distinct clustering between the treated and control groups, confirming a consistent and reproducible transcriptomic response to GF exposure. Gene Ontology (GO) enrichment analysis (Figure 2f) revealed that the DEGs were predominantly associated with ribosome‐related functions, including “protein‐containing complex,” “rRNA binding,” “ribonucleoprotein complex,” and “structural constituent of ribosome.” Kyoto Encyclopedia of Genes and Genomes (KEGG) pathway analysis (Figure 2g) identified “ribosome” as the most significantly enriched pathway, suggesting that GF may impair bacterial protein synthesis through ribosomal interference. Together, these results provide strong evidence that GF exerts antibacterial activity by targeting the ribosome and interfering with essential intracellular translation machinery. To verify the reliability of the transcriptomic results, we conducted qPCR analysis on selected upregulated and downregulated DEGs. The qPCR results were consistent with the RNA‐seq data (Figure 2h), confirming the reliability of the transcriptomic findings. Based on GO annotations, we further analyzed the functional roles of DEGs, with detailed gene information provided in Table S1. Following GF treatment, several ribosome‐associated genes—including rpsE (Log_2_FC −5.69), rplR (−5.80), and rpsN (−5.94)—were significantly downregulated. These genes encode essential components of the 30S and 50S ribosomal subunits, and their reduced expression suggests a disruption of ribosomal structure and function. In addition, the gene encoding FadA (C7Y58_RS04385), an essential virulence factor of F. *nucleatum* that promotes adhesion and invasion of epithelial and endothelial cells [58], was also downregulated after GF exposure. This finding indicates that GF may attenuate the pathogenic potential of F. *nucleatum* by suppressing the expression of its virulence genes. Conversely, several genes (C7Y58_RS01520, C7Y58_RS04030, C7Y58_RS08875 and pfkB) involved in bacterial stress response and metabolic adaptation were upregulated following treatment. These include genes encoding a drug/metabolite transporter (DMT) family, sensor histidine kinase, L‐lactate dehydrogenase, and 1‐phosphofructokinase. These proteins are implicated in toxin efflux, environmental signal sensing, and energy metabolism regulation [59, 60, 61, 62]. Upregulated expression of these genes suggests that F. *nucleatum* activates compensatory mechanisms in response to GF‐induced stress, including metabolic reprogramming and enhanced environmental adaptability. Collectively, these changes demonstrate that GF may trigger a broad and coordinated metabolic response in F. *nucleatum*.

Transcriptomic analysis indicated that the bacterial ribosome is a key node in the mechanism of GF. However, transcriptomic data alone cannot distinguish whether this inhibitory effect is an indirect consequence of membrane disruption, or whether GF directly targets the ribosome. As the ribosome is a major antibiotic target, the extensive use of antibiotics has already driven resistance in clinically relevant pathogens via strategies including efflux pumps and ribosomal mutations or modifications, thereby limiting therapeutic efficacy [63]. Given that positively charged GF can be electrostatically attracted to the negatively charged ribosome, we hypothesized that GF exerts its intracellular antibacterial effects by binding directly to rRNA at multiple sites. Such a non‐typical binding pattern could enable GF to bypass conventional antibiotic resistance mechanisms. To validate this hypothesis, we performed molecular docking and molecular dynamics simulations to investigate the potential interaction modes between GF and the bacterial ribosome at the atomic level and to identify its possible binding sites.

**FIGURE 3:**
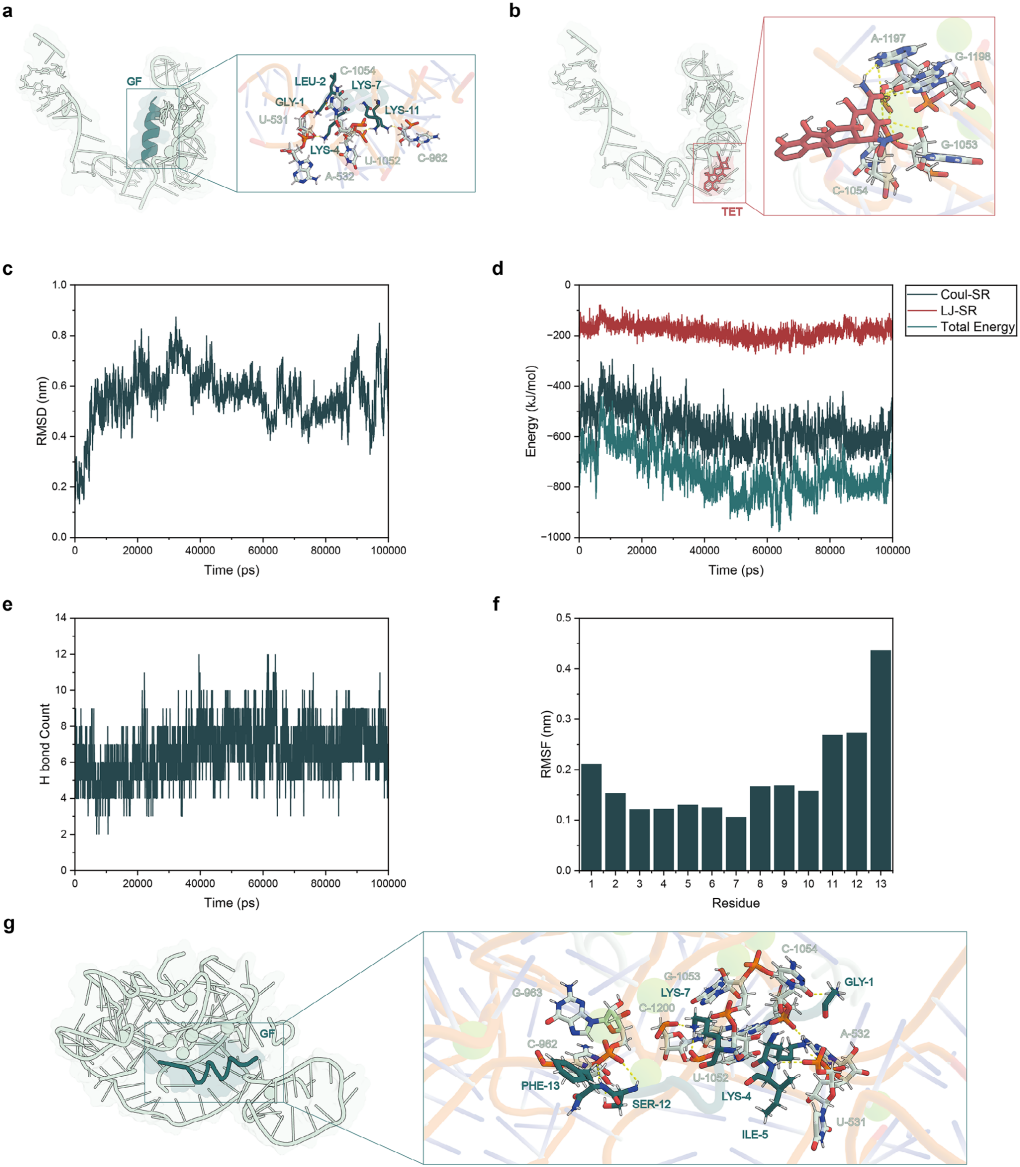
Molecular docking and dynamics simulation. (a) Binding mode of GF at the A‐site of the bacterial 30S ribosomal subunit. (b) Binding mode of tetracycline at the A‐site. (c) RMSD analysis. (d) Interaction energy analysis between GF and the A‐site. (e) Timeline representation of hydrogen bonds. (f) RMSF analysis. (g) Representative snapshot of GF bound at the A‐site, with key hydrogen bonds and relevant residues annotated.

Due to the high sequence and structural conservation of ribosomal RNA, the ribosome serves as a critical target for antimicrobial therapy [64]. The A‐site functions as the binding site for aminoacyl tRNA [65], while the peptide exit tunnel (PET) provides the essential pathway through which newly synthesized polypeptides exit [66, 67]. Tetracyclines and macrolides, commonly used in the clinical management of periodontitis [68, 69, 70], inhibit protein synthesis by binding to the A‐site and PET, respectively [66, 71]. Here, we selected the A‐site and PET as representative targets to explore the potential interactions between GF and the ribosome. Molecular docking results (Table S2) suggested that GF has a higher binding affinity to the ribosomal A‐site compared to the PET site, suggesting that its intracellular antibacterial activity is mainly driven by interactions with the A‐site. Therefore, we focused our subsequent analyses on the A‐site. Further refinement of the docking results (Table S2) yielded a stable and structurally reasonable GF‐A‐site binding model, which provided a reliable starting structure for molecular dynamics simulations. To evaluate the relative binding strength of GF, we compared it with tetracycline, a known A‐site inhibitor. The docking models (Figure 3a,b) revealed that GF engages the A‐site in a binding region distinct from that of tetracycline. Moreover, the docking results (Table 1) showed that GF had a lower HADDOCK score, suggesting a more stable binding configuration at the A‐site. This stability was largely attributed to stronger electrostatic and van der Waals interactions. Collectively, these findings indicate that GF adopts a unique binding mode at the ribosomal A‐site and is predicted to form more stable interactions than tetracycline.

**TABLE 1:** HADDOCK scores for GF and tetracycline bound at the ribosomal A‐site.

	HADDOCK score	RMSD	Vdw energy	Electrostatic energy	Desolvation energy	Restraint's violation energy	Buried Surface Area	Z‐Score
GF	−76.9 ± 2.4	1.1 ± 0.1	−49.2 ± 1.6	−318.1 ± 18.9	4.1 ± 1.4	0.2 ± 0.1	1280.4 ± 36.9	−1.5
tetracycline	−38.1 ± 0.7	1.3 ± 0.0	−19.2 ± 1.0	−209.9 ± 4.6	2.0 ± 0.2	0.7 ± 0.1	488.5 ± 11.5	−1.6

To assess the dynamic stability of the GF‐A‐site complex, we performed MD simulations. The results (Figure 3c) showed that after initial fluctuations, the root mean square deviation (RMSD) of the system gradually stabilized, indicating that the complex maintains its overall structural integrity during the simulation. Energy analysis (Figure 3d) further revealed that Coulombic short‐range (Coul‐SR) interaction contributes predominantly to the binding, while a substantial number of intermolecular hydrogen bonds remained stable throughout the simulation (Figure 3e). The root mean square fluctuation (RMSF) profile (Figure 3f) revealed that the central lysine residues of GF (Lys 4 and Lys 7) exhibited low fluctuation values, indicating stable interactions with the ribosome and highlighting them as potential binding hot spots. In contrast, residues located at the C‐terminus showed higher flexibility. These findings suggest that GF establishes a robust interaction network at the A‐site, ensuring stable binding of the complex. Moreover, representative equilibrium conformations extracted from the stable trajectory segment (Figure 3g) provide direct structural evidence supporting the stability of GF at the A‐site.

Cell‐free protein synthesis assay was performed to validate the inhibitory effect of GF on ribosomal translation predicted by molecular simulations (Figure S2). When 4 µm GF was added, DHFR translation was partially inhibited, whereas 8 µm GF almost entirely suppressed protein synthesis. This result provides direct experimental evidence that GF can interfere with ribosomal protein translation in a dose‐dependent manner.

In summary, the antimicrobial peptide GF exhibited strong bactericidal activity against F. *nucleatum*. Beyond its previously known membrane‐disrupting effect, this study provides the first evidence that GF can also target the ribosome through a binding mode distinct from conventional antibiotics, thereby interfering with bacterial protein synthesis. This dual mode of action—membrane disruption and ribosome targeting—may enhance its therapeutic potential in the management of infectious periodontitis.

Despite the promising antibacterial ability of GF, its clinical application is limited by several challenges, including enzymatic degradation, rapid clearance, and poor stability in complex biological environments [72, 73]. These limitations are particularly pronounced in the oral cavity, where fluctuating pH and high protease levels can significantly compromise its bioavailability and therapeutic efficacy. To tackle these problems, we developed a pH‐responsive MOF‐based delivery platform designed to protect GF from early degradation and enable targeted release in acidic inflammatory microenvironments. Additionally, this platform allows for the co‐delivery of bioactive molecules with anti‐inflammatory and osteogenic properties, achieving a multifaceted therapeutic effect that includes antibacterial, anti‐inflammatory, antioxidant, and bone‐regenerative outcomes. This integrated strategy offers a promising therapeutic approach for managing infectious periodontal diseases.

### 2.2 Synthesis and Characterization of M, I@M, G@M, and IG@M

Previous researches have demonstrated that Irisin exhibits strong anti‐inflammatory and antioxidant effects in inflammatory environments [34, 74]. Meanwhile, increased levels of proteases have been reported in the gingival crevicular fluid (GCF) and saliva of periodontitis patients [75, 76], which may impair the bioactivity of both Irisin and GF. To address this, we employed NH_2_‐MIL‐101 as a protective carrier to enhance drug stability and achieve targeted release. Based on this strategy, we developed four functional materials: unloaded NH_2_‐MIL‐101 (M), NH_2_‐MIL‐101 loaded with Irisin (I@M), NH_2_‐MIL‐101 loaded with GF (G@M), and NH_2_‐MIL‐101 co‐loaded with both agents (IG@M). These materials were systematically characterized for their physicochemical properties and biocompatibility.

NH_2_‐MIL‐101 was synthesized according to a previously reported procedure [77]. The structure was confirmed by Fourier‐transform infrared spectroscopy (FTIR, Figure S3a). The peak at 767 cm^−1^ was attributed to the stretching vibration of the O–Fe–O bond; the peak at 1387 cm^−1^ indicated the presence of −COO− groups; peaks at 1587 and 1687 cm^−1^ were associated with C = O and C = N bonds, respectively; and the peak at 3431 cm^−1^ corresponded to N–H stretching vibrations. Further confirmation was provided by X‐ray diffraction (XRD, Figure S3b), which showed distinct diffraction peaks at 5.18°, 8.53°, 9.08°, 10.33°, and 16.47°. These findings align with previous reports [78, 79], verifying the successful synthesis of NH_2_‐MIL‐101.

**FIGURE 4:**
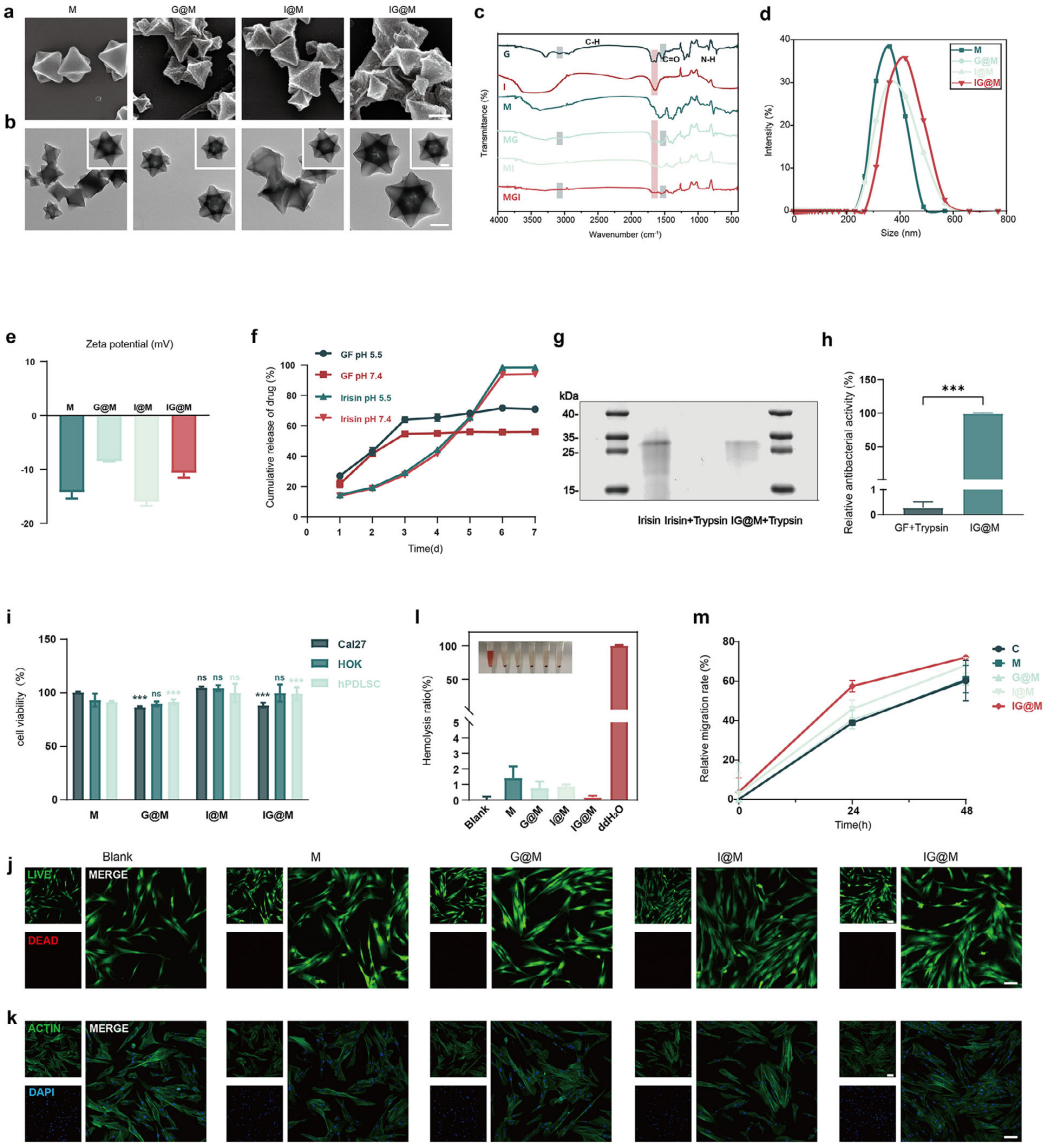
Construction, characterization, and biocompatibility assessment of the composite systems. (a) SEM images of M, G@M, I@M, and IG@M (scale bar: 200 nm). (b) TEM images (scale bars: main image, 200 nm; inset, 100 nm). (c) FTIR spectra. (d) DLS results. (e) Zeta potential measurements. (f) Release profiles of GF and Irisin from IG@M. (g) SDS–PAGE comparing Irisin, trypsin‐treated Irisin, and IG@M. (h) Relative antibacterial activity of GF and IG@M after trypsin treatment, normalized to untreated GF. (i) CCK‐8 analysis of HOK, Cal27, and hPDLCs at 48 h. (j) Live/dead fluorescence microscopy images of hPDLCs at 24 h (scale bar: 100 µm). (k) Cytoskeleton staining of hPDLCs at 24 h (scale bar: 100 µm). (l) Hemolysis assay. m) Statistical analysis of hPDLCs cell migration rates. Data in (e), (f), (h), (i), (l) and (m) are presented as means ± SD (n = 3). ns *p* > 0.05, ***p < 0.001.

To obtain the composite systems, Irisin and GF were loaded into NH_2_‐MIL‐101. Energy‐dispersive X‐ray spectroscopy (EDS, Figure S3c) confirmed that the main elemental composition—C, N, O, and Fe—remained unchanged after loading. The elemental ratios showed no significant deviation from those of the unloaded MOF, indicating that the incorporation of Irisin and GF did not alter the overall composition of the framework. Scanning electron microscopy (SEM, Figure 4a) showed that all four particle types maintained a regular octahedral morphology. However, particles loaded with Irisin and/or GF exhibited rougher surfaces and slightly increased diameters, suggesting the presence of these molecules on the particle surfaces and within the pores. Interestingly, transmission electron microscopy (TEM, Figure 4b) showed altered diffraction contrast in the MOF core of GF‐loaded samples (G@M and IG@M), implying potential changes in electron density or structural rearrangement caused by GF incorporation [80, 81]. By comparison, such changes were not observed in the Irisin‐only loaded sample (I@M). FTIR (Figure 4c) further confirmed the loading of drug molecules. In addition to the characteristic peaks of the MOF carrier, all three drug‐loaded materials exhibited a absorption band around 1650 cm^−1^, corresponding to the amide I band typical of the protein and peptide. For GF‐loaded samples, characteristic peaks corresponding to GF at around 1520 and 3080 cm^−1^ were also observed. Dynamic light scattering (DLS, Figure 4d) showed that the diameter of NH_2_‐MIL‐101 was 352.6 ± 48.45 nm. A slight increase in particle size was observed after drug loading, consistent with the SEM results. This nanoscale size facilitates retention within the periodontal pocket, potentially enhancing local drug concentrations [82, 83]. Zeta potential measurements (Figure 4e) revealed that the unloaded NH_2_‐MIL‐101 carried a surface charge of −14.20 mV. The loading of positively charged peptide GF increased the zeta potential to −8.45 mV. Irisin loading resulted in a potential of −16.03 mV. The dual‐loaded IG@M composite presented a zeta potential of −10.64 mV. The overall negative surface charge promotes electrostatic interaction with positively charged inflammatory sites, aiding in localized retention [84]. These alterations in particle size and surface charge provide consistent evidence for successful drug incorporation.

NH_2_‐MIL‐101 possesses a large specific surface area and suitable pore size, making it well‐suited for efficient loading of peptides and proteins [49]. In the IG@M composite, the loading efficiencies of Irisin and GF were 84.94 ± 0.02% and 71.78 ± 4.09%, respectively. To investigate the release behavior of IG@M, drug release profiles were assessed under neutral (pH 7.4) and mildly acidic (pH 5.5) conditions to simulate the environments of healthy tissue and periodontitis lesions (Figure 4f). Under neutral condition, GF showed an initial release of 55% within the first 3 days, after which the cumulative release tended to plateau. In contrast, Irisin was released more gradually, reaching 95% by day 7. In acidic condition, GF exhibited a faster release, with cumulative amounts increasing to 65% at day 3 and 70% at day 7. Irisin showed a similar release pattern under both pH conditions, reaching approximately 98% at day 7. This behavior may be attributed to the distinct interactions between the cargo molecules and the MOF scaffold, which could be explored in future studies.

To assess whether the IG@M composite could resist proteolytic degradation, we examined the integrity of Irisin after trypsin treatment. Coomassie Brilliant Blue staining (Figure 4g) revealed a distinct Irisin band at approximately 25–35 kDa, consistent with previous reports [85]; the band completely disappeared when free Irisin was exposed to trypsin, indicating enzymatic degradation. In contrast, a visible Irisin band remained in the IG@M group, suggesting that the composite can protect Irisin from proteolysis. Moreover, compared with trypsin‐treated free GF, the trypsin‐treated IG@M sample retained over 99% of its antibacterial activity (Figure 4h). These findings indicate that IG@M effectively protects both Irisin and GF from protease‐induced degradation.

These results demonstrate the pH‐responsive release capability of IG@M, aligning with the acidic microenvironment of periodontitis. This may facilitate targeted drug delivery to inflamed sites while minimizing off‐target effects in healthy tissue. Moreover, the sequential release of GF and Irisin corresponds with the staged treatment needs of periodontitis, indicating the system's potential for synergistic and precise therapeutic applications.

### 2.3 Biocompatibility Evaluation

To evaluate the biocompatibility of NH_2_‐MIL‐101 as a drug delivery carrier in oral environment, CCK‐8 assays were conducted on human oral keratinocytes (HOK), the human oral squamous cell carcinoma cell line (Cal27), and hPDLCs cells across a range of concentrations. As shown in Figure S4a, NH_2_‐MIL‐101 exhibited no significant cytotoxicity at concentrations below 100 µg/mL, with cell viability remaining above 90%. This finding was supported by live/dead staining of hPDLCs (Figure S4b) and hemolysis assays (Figure S4c), confirming the material's good biosafety for potential oral applications.

Following the confirmation, further biocompatibility assessments were performed on the drug‐loaded materials. CCK‐8 assays (Figure 4i) and live/dead staining (Figure 4j) demonstrated that all four material treatment groups maintained high cell viability (>85%), indicating favorable cytocompatibility. Fluorescent staining of the cytoskeleton (Figure 4k) showed well‐preserved structure in hPDLCs across all treatment groups. In addition, Reactive oxygen species (ROS) staining (Figure S5a) revealed no increase in intracellular reactive oxygen species, suggesting that the materials did not induce oxidative stress. Given that local inflammation in periodontitis can increase drug absorption by promoting capillary dilation and vascular permeability, we assessed the hemolytic potential of each material (Figure 4l). All samples exhibited hemolysis rates under 5%, in accordance with the ISO 10993‐4 standard. To preliminarily explore the materials' potential to enhance tissue regeneration, a wound healing assay was conducted to assess their impact on hPDLCs migration (Figure S5b). Quantitative analysis (Figure 4m) showed that Irisin‐loaded materials (I@M and IG@M) promoted cell migration, consistent with previous findings [35].

In summary, the developed composite materials demonstrate excellent physicochemical stability and biosafety, supporting their potential application in antibacterial, anti‐inflammatory, and tissue regeneration therapies.

### 2.4 In Vitro Antibacterial Activity of the Composite Delivery System

Periodontitis is a chronic inflammatory disease triggered by multiple factors, with bacterial infection recognized as the primary initiator of disease onset and progression [86, 87]. Therefore, the effective suppression of pathogenic bacterial proliferation and biofilm formation is essential for halting disease advancement. Based on the confirmed antibacterial activity of GF against F. *nucleatum*, the composite delivery system was further evaluated for its ability to inhibit colonization and biofilm formation of representative periodontal pathogens, including S. *aureus*, F. *nucleatum*, and A. *actinomycetemcomitans* (A.a).

**FIGURE 5:**
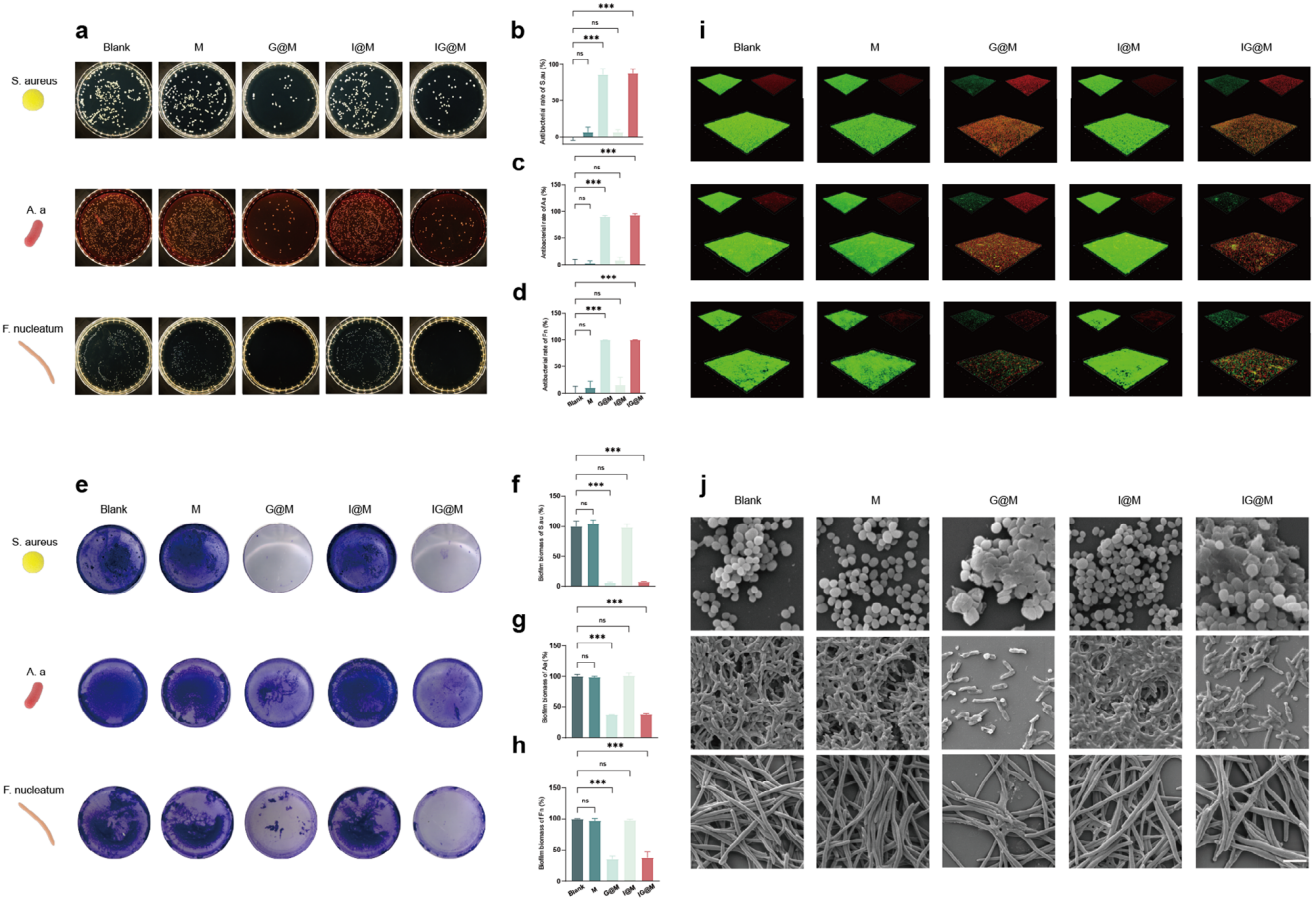
In vitro antibacterial performance of the composite systems. (a–d) Images of colonies on agar plates and corresponding antibacterial rate of M, G@M, I@M, and IG@M against S. *aureus*, A. *actinomycetemcomitans*, and F. *nucleatum*. (e–h) Inhibition effects and statistical analysis of S. *aureus*, A. *actinomycetemcomitans*, and F. *nucleatum* biofilms by the composite systems. (i) 3D reconstruction of S. *aureus*, A. *actinomycetemcomitans*, and F. *nucleatum* biofilms after treatment with M, G@M, I@M, or IG@M. (j) Representative SEM images of biofilms (scale bar: 2 µm). Quantitative data are presented as means ± SD (n = 3). ns *p* > 0.05, ***p < 0.001.

As shown in the Figure 5a–d, neither the M group nor the I@M group exhibited notable antibacterial activity compared to the control. By comparison, both the G@M and IG@M treatment groups demonstrated significant antibacterial effects. Specifically, G@M and IG@M treatments achieved significantly higher bacterial inhibition rates (S. *aureus* >85%, A.a >90%, and F. *nucleatum* >99%), whereas the M and I@M groups remained comparable to the control. The formation of periodontal biofilms poses a significant challenge to antimicrobial therapy, as bacteria within biofilms possess markedly higher resistance to antibiotics than those in planktonic form. Moreover, the negatively charged matrix of biofilms hinders the penetration of cationic agents [88, 89]. In this study, the anionic MOF system was designed to facilitate diffusion into the biofilm and release the cationic peptide GF in response to the acidic microenvironment at inflamed sites. This strategy enables targeted disruption of the biofilm structure. Crystal violet staining was applied to assess the inhibitory effects of each group on early biofilm formation. As shown in Figure 5e, the control, M and I@M groups exhibited deep purple appearance, indicating the presence of dense biofilms. In contrast, the G@M and IG@M treatment groups showed ruptured biofilm structure and noticeably lighter staining. Quantitative analysis (Figure 5f–h) confirmed a significant reduction in biomass for both treatment groups (S. *aureus* <10%, A.a <40%, and F. *nucleatum* <40%), suggesting effective inhibition of initial bacterial adhesion. Confocal laser scanning microscopy (CLSM) was employed to examine the 3D structure of mature biofilms following treatment. As shown in Figure 5i, the GF‐containing systems significantly reduced biofilm thickness and disrupted structural integrity. These findings further confirm that both G@M and IG@M possess the ability to degrade mature biofilms. SEM was used to examine bacterial morphology within biofilms following different treatments (Figure 5j). In the control, M, and I@M groups, bacteria maintained intact surface structures with no evident membrane damage. In contrast, bacteria in the G@M and IG@M groups exhibited disrupted membranes and collapsed morphology, indicating that GF exerted its antibacterial effect by damaging bacterial membranes within the biofilm. These findings confirm the contribution of GF within the composite system.

In summary, the IG@M composite delivery system demonstrated a multifaceted antibacterial mechanism in vitro. Given that periodontitis arises from a complex interaction between bacterial infection and host immune dysregulation, the system's rapid and effective antibacterial activity at the early stage lays a solid foundation for subsequent anti‐inflammatory, antioxidative, and osteogenic therapeutic responses.

### 2.5 Anti‐Inflammatory and Antioxidant Effects

**FIGURE 6:**
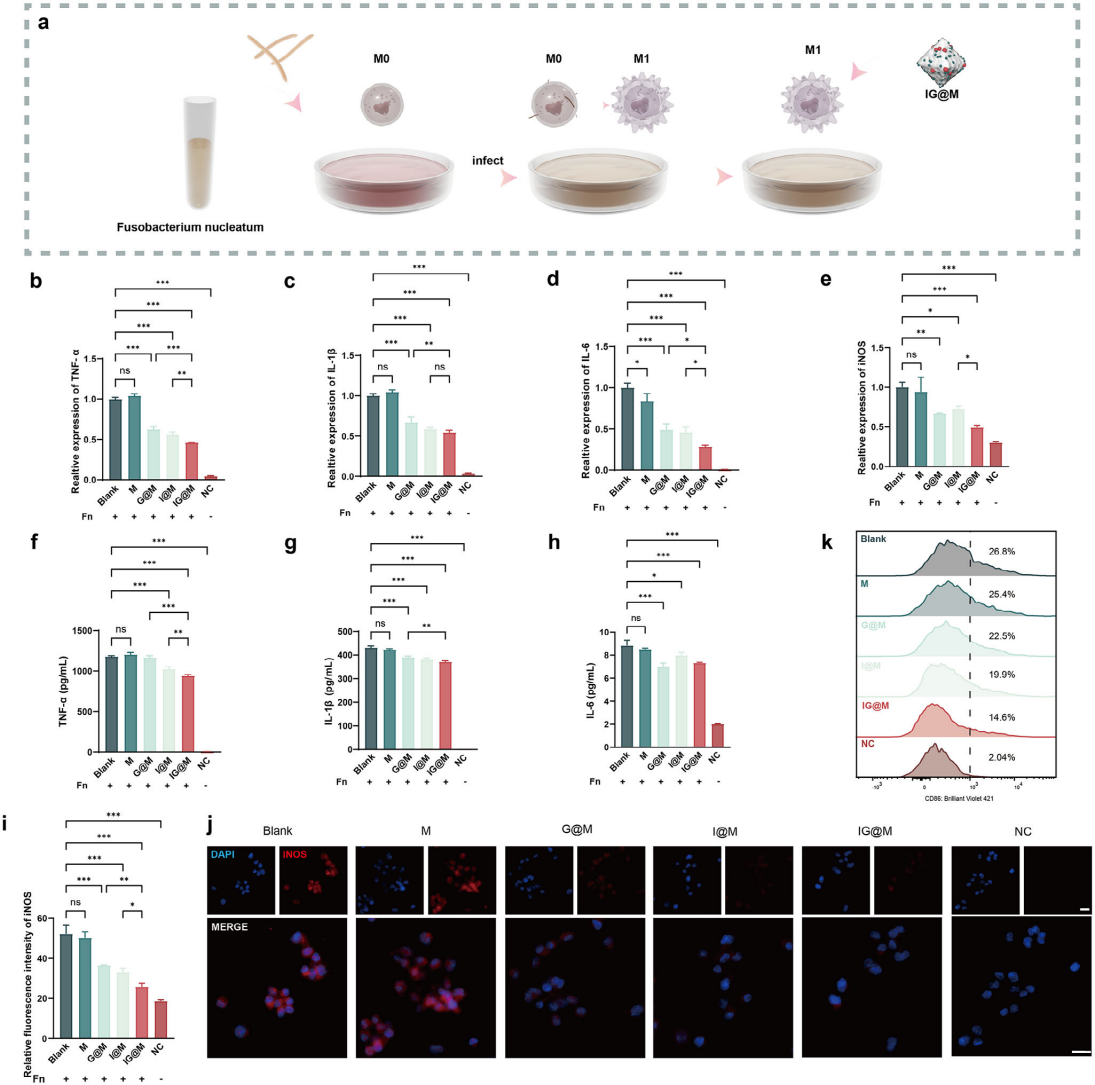
Evaluation of the anti‐inflammatory effects of the composite systems. (a) Schematic illustration of the F. *nucleatum*‐infected THP‐1 cell model. (b–e) qPCR analysis of TNF‐α, IL‐1β, IL‐6, and iNOS expression. (f–h) ELISA quantification of TNF‐α, IL‐1β, and IL‐6 secretion. (i,j) Representative fluorescence microscopy images and statistical analysis of iNOS (scale bar: 25 µm). (k) Flow cytometric analysis of CD86 expression. Quantitative data are presented as mean ± SD (n = 3). ns *p* > 0.05, *p < 0.05, **p < 0.01, ***p < 0.001.

During the progression of periodontitis, the interplay between host immune responses and microbial dysbiosis contributes to continuous periodontal tissue destruction [90]. A key pathological feature of chronic periodontitis is the failure to resolve inflammation in a timely manner [4]. Researches indicated that macrophage infiltration is markedly increased in the periodontal tissues of affected individuals [91]. Among them, M1 phenotype polarization is critical for maintaining a pro‐inflammatory environment. M1 macrophages produce elevated amounts of pro‐inflammatory cytokines and promote osteoclast differentiation, thereby exacerbating tissue breakdown [92]. F. *nucleatum* has been shown to drive M1 polarization and can even invade and persist within macrophages, contributing to prolonged inflammatory responses and disease progression [93, 94]. To replicate the pathogenic inflammatory microenvironment of periodontitis, we established a co‐culture model of F. *nucleatum*‐infected acute monocytic leukemia cells (THP‐1)‐derived macrophages to access the anti‐inflammatory efficacy of the composite delivery system (Figure 6a).

TNF‐α, IL‐1β, and IL‐6 play pivotal roles in amplifying the inflammatory response and promoting osteoclast differentiation, thereby accelerating alveolar bone resorption [95, 96]. In addition, bacteria‐induced iNOS expression may contribute to tissue damage through excessive nitric oxide (NO) production [97]. These mediators represent key therapeutic targets in the treatment of periodontitis. qPCR analysis (Figures 6b–e) revealed that F. *nucleatum* infection significantly upregulated the mRNA expression of pro‐inflammatory cytokines TNF‐α, IL‐1β, IL‐6, and iNOS by approximately 20‐, 25‐, 100‐, and 3.3‐fold, respectively, indicating activation of inflammatory signaling pathways and sustained inflammatory responses. The M group showed no apparent anti‐inflammatory activity. In contrast, G@M reduced TNF‐α, IL‐1β, IL‐6, and iNOS expression by approximately 1.5–2.0‐fold, I@M decreased these cytokines by 1.4–2.2‐fold, and IG@M exerted the most pronounced inhibition (1.8–3.4‐fold), showing the best effects on TNF‐α and IL‐6 expression compared to G@M and I@M, indicating a broader overall inhibitory effect of the co‐delivery system. Consistent with the qPCR results, Enzyme‐Linked Immunosorbent Assay (ELISA) measurements (Figure 6f–h) confirmed that all three treatment groups, G@M, I@M, and IG@M, markedly reduced the secretion levels of TNF‐α, IL‐1β, and IL‐6. Immunofluorescence staining and quantification of iNOS expression (Figure 6i,j) also showed clear reductions following treatment with G@M, I@M, or IG@M, indicating effective suppression of inflammatory mediator production at the protein level. To assess the influence of the materials on macrophage polarization, CD86, a surface marker of M1 macrophages, was analyzed using flow cytometry (Figure 6k). All three treatments led to decreased CD86 expression, confirming that the drug‐loaded MOF systems effectively inhibited M1‐type polarization of macrophages.

**FIGURE 7:**
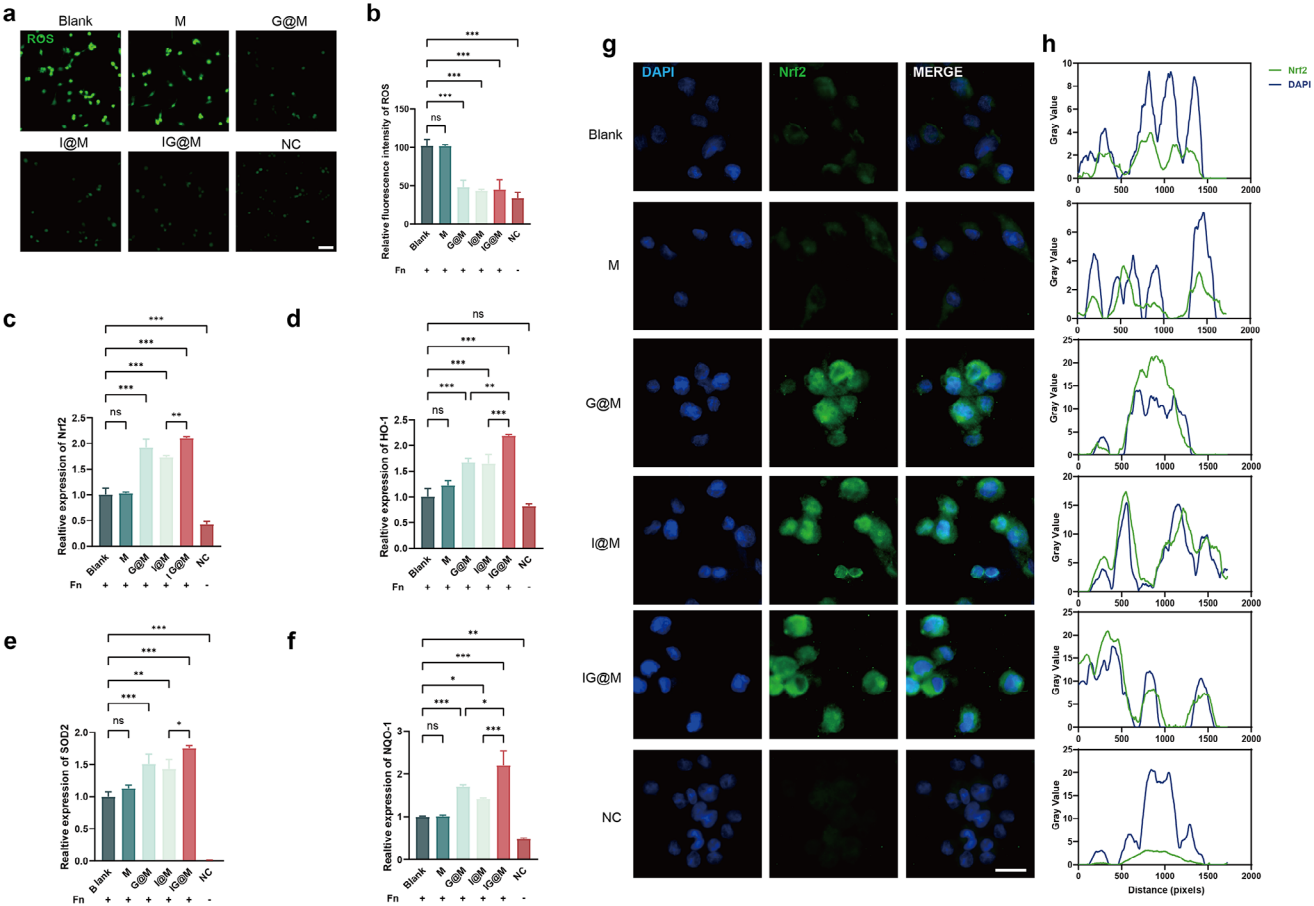
Evaluation of the antioxidant activity of the composite systems. (a,b) ROS detection of intracellular reactive oxygen species levels (scale bars: 100 µm). (c–f) qPCR analysis of Nrf2, HO‐1, SOD2, and NQO1 expression. (g,h) Fluorescence microscopy images of Nrf2 and its colocalization with DAPI (scale bars: 25 µm). Quantitative data are presented as mean ± SD (n = 3). ns *p* > 0.05, *p < 0.05, **p < 0.01, ***p < 0.001.

Following pathogen internalization, neutrophils activate NADPH oxidase to produce large amounts of ROS [98]. While ROS are essential for host defense, their overproduction can directly damage periodontal tissues and stimulate osteoclast differentiation, leading to alveolar bone resorption [87, 99]. Therefore, regulating ROS levels is also essential for the effective management of periodontitis. DCFH‐DA probe was applied to assess the intracellular ROS levels. As shown in the Figure 7a,b, F. *nucleatum* infection markedly increased green fluorescence intensity, indicating elevated ROS production. In contrast, treatment with G@M, I@M, and IG@M significantly decreased ROS levels, reducing the fluorescence intensity to approximately 50% of that observed in F. *nucleatum* group. During the progression of periodontitis, nuclear factor erythroid 2‐related factor 2 (Nrf2) translocates to the nucleus and regulates the transcription of antioxidant genes, thereby reducing inflammatory signaling and oxidative damage [100]. HO‐1, SOD2, and NQO1 were selected as representative downstream targets of Nrf2 due to their critical roles in antioxidant defense. HO‐1 catalyzes the conversion of heme into bilirubin and directly suppresses pro‐inflammatory cytokines [101]. NQO1 acts as an endogenous coenzyme Q reductase to maintain cellular antioxidant protection [102], while SOD2 serves as a key mitochondrial enzyme that detoxifies superoxide radicals [103]. As confirmed by qPCR (Figure 7c–f), G@M, I@M, and IG@M upregulated the expression of Nrf2, HO‐1, SOD2, and NQO1, consistent with the observed reduction in pro‐inflammatory cytokines. Further immunofluorescence staining (Figure 7g,h) confirmed that all three treatments enhanced Nrf2 expression and promoted its nuclear translocation, suggesting activation of the cellular antioxidant defense mechanism. Previous studies have revealed the immunomodulatory and antioxidant effects of Irisin [104]. Notably, GF did not exhibit significant anti‐inflammatory or antioxidant effects in the LPS‐stimulated macrophage model (Figure S6a–f). This observation suggests that GF may exert its effects through an intracellular antibacterial mechanism or by selectively inhibiting F. *nucleatum*‐induced inflammatory pathways. These potential mechanisms will be further investigated in future studies.

Overall, IG@M exhibited anti‐inflammatory and antioxidant effects in the F. *nucleatum*‐induced inflammatory model, thereby establishing a favorable microenvironment for subsequent osteogenic regeneration.

### 2.6 Evaluation of Osteogenic Efficacy

Early intervention targeting bacterial infection, inflammation, and oxidative stress is essential to stabilize the lesion and prevent further tissue damage in the treatment of periodontitis. In the later stages, promoting bone regeneration becomes equally important for restoring periodontal structure [105, 106]. Therefore, a staged therapeutic strategy that transitions from infection control to tissue repair is critical for achieving optimal outcomes. To investigate the osteogenic capability of the composite systems, we evaluated the impact of the materials on osteogenesis‐related biological responses in hPDLCs at multiple time points.

**FIGURE 8:**
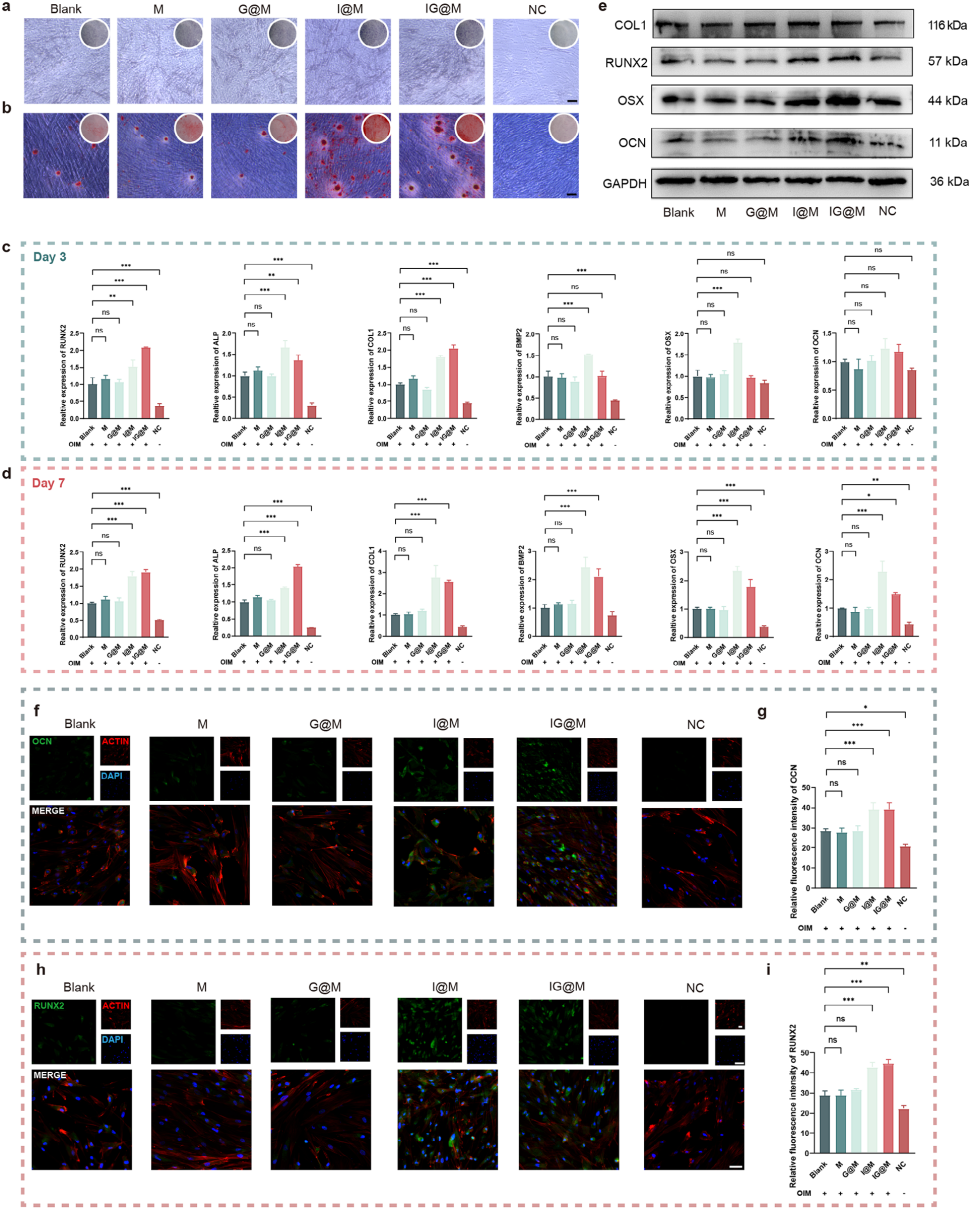
Evaluation of the osteogenic performance of the composite systems. (a) ALP staining images of different treatment groups (scale bar: 100 µm). (b) ARS staining images (scale bar: 100 µm). (c,d) qPCR analysis of RUNX2, ALP, COL1, BMP2, OSX, and OCN expression on (c) day 3 and (d) day 7. (e) Western blot analysis of RUNX2, OSX, COL1, and OCN. (f,g) Fluorescence images and statistical analysis of OCN, and (h,i) RUNX2 (scale bar: 25 µm). ns *p* > 0.05, *p < 0.05, **p < 0.01, ***p < 0.001 (n = 3).

Alkaline phosphatase (ALP) is an early indicator of osteogenic activity [107]. ALP staining (Figure 8a) revealed more intense coloration in the I@M and IG@M groups, suggesting enhanced early‐stage osteogenic differentiation. In contrast, the formation of mineralized nodules reflects the later stages of osteoblast maturation. Alizarin Red S (ARS) staining (Figure 8b) showed a greater number of mineralized nodules in both groups after induction, indicating superior mineralization capacity during the late phase of osteogenesis. Among various transcriptional and epigenetic regulators, RUNX2 and OSX serve as the core transcription factors driving osteogenic differentiation, ALP and COL1 represent the predominant osteogenic markers, BMP functions as an essential developmental factor orchestrating the osteogenic program, and OCN indicates the terminal differentiation phase of osteoblasts [108]. qPCR analysis (Figure 8c,d) demonstrated that both I@M and IG@M groups exhibited elevated expression of osteogenic genes, indicating their osteoinductive potential. Following osteogenic induction, all groups showed markedly increased expression of osteogenic markers compared to the non‐induced negative control. Specifically, in the Irisin‐loaded groups (I@M and IG@M), RUNX2 expression was consistently upregulated at both day 3 and day 7 (I@M: 3 d 1.53 ± 0.19, 7 d 1.80 ± 0.14; IG@M: 3 d 2.08 ± 0.03, 7 d 1.91 ± 0.06), suggesting its involvement in directing hPDLC osteogenic differentiation. For early‐ to mid‐stage markers such as ALP (I@M: 3 d 1.66 ± 0.16, 7 d 1.41 ± 0.03; IG@M: 3 d 1.37 ± 0.12, 7 d 2.04 ± 0.05), COL1 (I@M: 3 d 1.79 ± 0.05, 7 d 2.76 ± 0.56; IG@M: 3 d 2.04 ± 0.11, 7 d 2.55 ± 0.08), BMP2 (I@M: 3 d 1.52 ± 0.01, 7 d 2.43 ± 0.36; IG@M: 3 d 1.02 ± 0.12, 7 d 2.10 ± 0.27), and OSX (I@M: 3 d 1.80 ± 0.08, 7 d 2.33 ± 0.16; IG@M: 3 d 0.98 ± 0.04, 7 d 1.78 ± 0.26), expression levels were generally higher on day 7 than on day 3, suggesting a time‐dependent enhancement of osteogenic activity. As a late‐stage marker, OCN showed minimal change on day 3 but was increased by day 7 (I@M: 3 d 1.23 ± 0.18, 7 d 2.29 ± 0.37; IG@M: 3 d 1.17 ± 0.14, 7 d 1.49 ± 0.06). These findings support the dynamic regulatory capacity of the composite system throughout the osteogenic process. Western blot analysis (Figure 8e) revealed that treatment with I@M and IG@M increased the protein expression levels of RUNX2, OSX, COL1, and OCN, indicating their positive regulatory effects on osteogenesis at the protein level. Immunofluorescence staining (Figure 8f–i) also revealed that both treatments significantly enhanced the intracellular expression of RUNX2 and OCN, further supporting their role in promoting osteogenic differentiation.

In summary, both I@M and IG@M enhanced the expression of osteogenic proteins and promoted mineralization, demonstrating strong bone‐forming potential. Given the combined antibacterial, anti‐inflammatory, and antioxidant properties of IG@M, these results support its promise as a comprehensive therapeutic agent for the treatment of infectious periodontitis.

### 2.7 In Vivo Therapeutic Efficacy of IG@M Against F. *nucleatum*‐Induced Periodontitis

**FIGURE 9:**
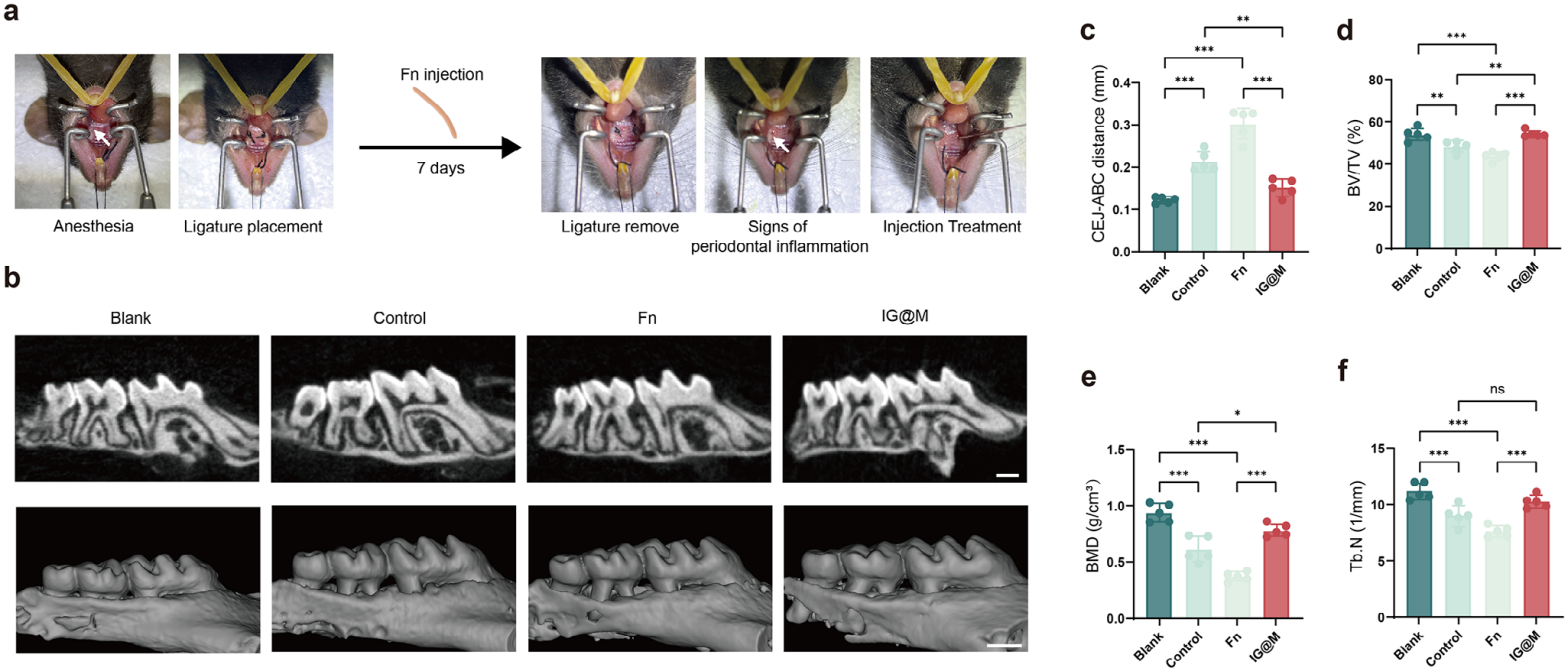
Therapeutic effects of IG@M in an infectious periodontitis model. (a) Schematic illustration of the experimental procedure. (b) Micro‐CT buccal‐palatal sectional views and 3D reconstructions of maxillary molars area (scale bar: 500 µm). (c) Quantitative analysis of the distance from the CEJ to the ABC. (d) Quantitative analysis of BV/TV. (e) Quantitative analysis of BMD. (f) Quantitative analysis of Tb.N. ns *p* > 0.05, *p < 0.05, **p < 0.01, ***p < 0.001 (n = 5).

Building on the demonstrated antibacterial and osteogenic potential of IG@M in vitro, we established a mouse model of infectious periodontitis by ligating the maxillary second molars and inoculating with F. *nucleatum*. Targeted treatments were applied locally at the lesion sites to evaluate the in vivo therapeutic efficacy. The experimental workflow is illustrated in the Figure 9a.

To assess the in vivo biosafety of IG@M, H&E staining was conducted on major organs, including the heart, liver, spleen, lungs, and kidneys (Figure S7b). Histological examination revealed negligible morphological or structural variations in any group after 4 weeks of treatment. These findings align with previous reports demonstrating the favorable biocompatibility and degradability of Fe‐based MOFs in vivo [109]. The organic ligands can be naturally metabolized under physiological conditions. Moreover, in contrast to Zn^2+^, Fe^3+^ released from Fe‐MOF degradation is less likely to induce metabolic imbalance or cytotoxicity [110], thereby avoiding accumulation or tissue injury in organs such as liver and kidney [111].

Micro‐computed tomography (micro‐CT) and 3D reconstruction images (Figure 9b) revealed significant alveolar bone loss around the second molars in the periodontitis group compared to healthy controls, which was further exacerbated by F. *nucleatum* infection. By comparison, mice treated with IG@M exhibited markedly reduced bone resorption. Quantitative analysis further confirmed this trend (Figure 9c–f): the vertical distance from the cementoenamel junction (CEJ) to the alveolar bone crest (ABC), bone volume ratio (BV/TV), bone mineral density (BMD), and trabecular number (Tb.N) were substantially decreased in both the periodontitis and infection groups but were notably restored following IG@M treatment. These results indicate that the composite delivery system effectively suppresses inflammation‐induced alveolar bone loss and supports bone tissue regeneration.

**FIGURE 10:**
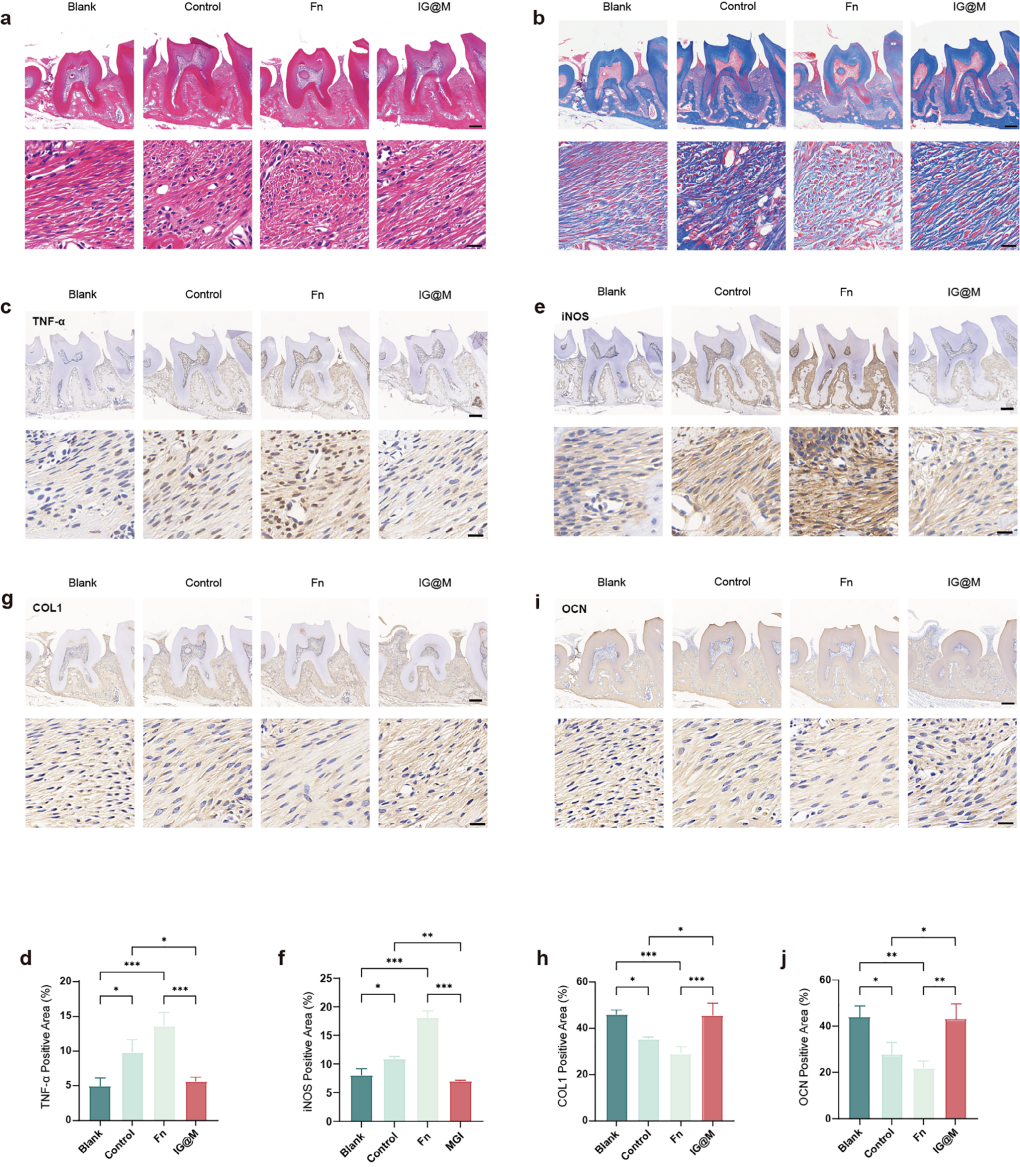
Histological and immunohistochemical analyses of IG@M in an in vivo model. (a) H&E staining. (b) Masson's trichrome staining. (c–j) Immunohistochemical staining and quantitative analysis of (c,d) TNF‐α, (e,f) iNOS, (g,h) COL1, and (i,j) OCN (scale bars: 200 µm for main images; 20 µm for magnified views). ns *p* > 0.05, *p < 0.05, **p < 0.01, ***p < 0.001 (n = 3).

To further confirm the therapeutic effects of IG@M, histological analysis of periodontal tissues was performed using H&E and Masson trichrome staining. H&E staining (Figure 10a) revealed that the healthy control group maintained intact epithelial attachment, low levels of inflammatory cell infiltration, and preserved alveolar crest structure. Conversely, the periodontitis and F. *nucleatum*‐infected groups showed epithelial detachment, extensive inflammatory infiltration, and alveolar bone destruction, indicating severe periodontal inflammation. In comparison, IG@M group showed partial restoration of epithelial attachment and a marked reduction in inflammatory infiltration. Masson trichrome staining (Figure 10b) provided further support for these observations. Collagen fibers in the periodontitis and F. *nucleatum* groups were reduced and disorganized, whereas IG@M group displayed more abundant and well‐aligned collagen deposition. These findings demonstrate that IG@M effectively alleviates inflammation and promotes periodontal tissue regeneration in a F. *nucleatum*‐induced periodontitis model. Giemsa staining was performed to detect bacteria in tissue sections (Figure S8). Due to the introduction of exogenous bacteria, abundant bacterial aggregates were observed in the periodontal tissues of the infection group, whereas no bacterial cells were detected in either the healthy control group or the ligature‐induced periodontitis group. In contrast, the IG@M group showed a pronounced reduction in bacterial presence, indicating that the composite exerts effective antibacterial activity in vivo. The local inflammatory microenvironment in periodontitis is a key contributor to alveolar bone resorption. TNF‐α plays a central role in promoting tissue damage and accelerating bone loss in periodontal lesions [112, 113]. Immunohistochemical (IHC) staining (Figure 10c,d) revealed elevated TNF‐α expression in the periodontitis group compared to the healthy control, with F. *nucleatum* infection further intensifying this inflammatory response. In contrast, the IG@M group showed a marked reduction in the positive staining area, suggesting that the MOF‐based system effectively mitigates local inflammation. iNOS‐mediated NO production could enhance osteoclast activity, thereby accelerating the progression of periodontitis [114]. The staining results (Figure 10e,f) revealed that the iNOS‐positive area in the IG@M group was significantly lower than that in the F. *nucleatum* and periodontitis groups, suggesting that the composite effectively alleviates oxidative stress associated with periodontitis. To evaluate the osteogenic potential of IG@M in the context of infectious periodontitis, we further assessed the expression of osteogenesis‐related markers, osteocalcin (OCN) and collagen type I (COL1) (Figure 10g–j). The results demonstrated that IG@M treatment significantly upregulated the expression of both markers. Quantitative analysis confirmed that their levels were higher in the IG@M group than in the periodontitis and infection groups. These findings indicate that, beyond its anti‐inflammatory effects, the IG@M actively contributes to periodontal tissue repair and bone regeneration.

## 3 Conclusion

This study focuses on key challenges in the treatment of periodontitis. To our knowledge, this is the first report revealing the intracellular mechanism of the antimicrobial peptide GF, including its ribosomal target that differs from that of tetracycline. In parallel, we developed a co‐delivery system based on NH_2_‐MIL‐101(Fe) to sequentially release GF and the adipomyokine Irisin. This system enables staged therapeutic effects: rapid early release of GF for infection control, mid‐phase anti‐inflammatory and antioxidant action through the synergistic action between GF and Irisin, and sustained Irisin release to promote bone regeneration, thus providing a “antimicrobial peptide–osteogenic protein” cascade strategy. Further studies, including long‐term biosafety assessment and pharmacokinetic testing, are expected before clinical application. Together, this study advances our understanding of GF's antibacterial mechanism and offers a novel, targeted approach to periodontitis therapy, offering strong potential for clinical translation.

## 4 Experimental Section

### 4.1 Materials

FeCl_3_⋅6H_2_O was purchased from Macklin Biochemical Technology Co., Ltd. (Shanghai, China). 2‐Aminoterephthalic acid (BDC‐NH_2_) and N,N‐dimethylformamide (DMF) were obtained from Aladdin Biochemical Technology Co., Ltd. (Shanghai, China). The antimicrobial peptide GF (sequence: GLFKIIKKIAKSF) and fluorescein isothiocyanate‐labeled GF (FITC‐GF) were synthesized by GenScript Biotech Corporation (Nanjing, China). Irisin was obtained from ABclonal Biotechnology Co., Ltd. (Wuhan, China).

### 4.2 Cell Culture

Cal27 (RRID: CVCL_1107) and THP‐1 (RRID: CVCL_0006) were purchased from the China Center for Type Culture Collection (CCTCC, Wuhan, China) in 2024. HOK were purchased from ScienCell Research Laboratories (USA) in 2023. All cell cultures were routinely tested and confirmed to be free of mycoplasma contamination. Cal27 cells were cultured in DMEM/F12 medium (Gibco, Scotland) containing 10% fetal bovine serum (FBS, Vazyme Biotech Co., Ltd., China) and 1% penicillin‐streptomycin (Beyotime, Shanghai, China). THP‐1 cells were maintained in RPMI 1640 medium containing 10% FBS and 1% penicillin‐streptomycin. HOK cells were cultured in DMEM medium containing identical supplements. All cells were incubated at 37 °C in a humidified atmosphere with 5% CO_2_.

### 4.3 Isolation and Culture of hPDLCs

This study was approved by the Ethics Committee of the Affiliated Stomatological Hospital of Nanjing Medical University (Approval No. PJ2023‐131‐001). All samples were collected from healthy premolars extracted for orthodontic purposes, with informed consent obtained from all participants or their guardians. Under sterile conditions, periodontal ligament tissue was carefully scraped from the root surface using a sterile scalpel. The tissue was then washed, centrifuged, and maintained in α‐MEM supplemented with 10% FBS and 1% penicillin‐streptomyc in a 5% CO_2_ atmosphere at 37°C. The culture medium was refreshed every 3–4 days, and the cells were passaged when reaching 70%–80% confluence. P3‐P6 cells were utilized for subsequent experiments.

### 4.4 Bacterial Strains and Cultivation Conditions

*Staphylococcus aureus* (ATCC 29213) was purchased from the China Center of Industrial Culture Collection (CICC, Beijing, China). *Fusobacterium nucleatum* (ATCC 25586) and *Aggregatibacter actinomycetemcomitans* (ATCC 43717) were provided by the Shanghai Bioresource Collection Center (SHBCC, Shanghai, China). Unless otherwise specified, all bacterial strains were cultured under the following conditions: S. *aureus* was grown on Luria‐Bertani (LB) agar plates at 37 °C under aerobic conditions. F. *nucleatum* and A. *actinomycetemcomitans* were cultured on Brain Heart Infusion (BHI) agar and blood agar plates, respectively at 37 °C in an anaerobic chamber (80% N_2_, 10% H_2_, 10% CO_2_) for 24–48 h. From each plate, a single colony was subsequently inoculated into the corresponding liquid medium and incubated at 37 °C until the bacterial cultures reached the logarithmic growth phase. The resulting bacterial suspensions were diluted to the concentration for subsequent experiments.

### 4.5 Antibacterial Activity of GF Against F. *nucleatum*

F. *nucleatum* was cultured anaerobically in BHI broth at 37°C until reaching the logarithmic growth phase. The bacterial suspension was then adjusted to 1 × 10^6^ CFU/mL. GF was serially diluted to concentrations ranging from 0.25 to 16 µm and added to a 96‐well plate. After adding 100 µL of the bacterial suspension, plates were incubated anaerobically at 37°C for 24 h. Bacterial growth was assessed by measuring the optical density at 600 nm (OD_600_) using a microplate reader (Spark, TECAN, Austria). MIC was defined as the minimal peptide concentration preventing visible bacterial growth. 10 µL samples from wells were plated onto BHI agar plates and incubated at 37°C for 48 h. MBC was determined as the lowest concentration that completely inhibited colony formation.

### 4.6 Cellular Internalization of GF by F. *nucleatum*

FITC‐labeled GF (concentration: 16 µm) was incubated with F. *nucleatum* suspension (1 × 10^8^ CFU/mL) under anaerobic conditions at 37°C. Samples were collected at 0, 10, 30, and 60 min, followed by staining with DAPI (1 µg/mL) for 10 min to visualize bacterial DNA. The fluorescence signals were observed using an upright fluorescence microscope (Leica DM6 B, Germany) to assess the internalization of GF and its interaction with F. *nucleatum* over time.

### 4.7 Prokaryotic Transcriptome Sequencing and Analysis

Samples from both GF‐treated group (16 µm GF incubated for 1 h) and control group (incubated with an equal volume of PBS) were collected by centrifugation to remove the culture medium, then immediately snap‐frozen in liquid nitrogen. Total RNA extraction and transcriptome sequencing were performed by Majorbio Bio‐Pharm Technology Co., Ltd. (Shanghai, China). RNA‐seq libraries were prepared using the Illumina TruSeq RNA Sample Preparation Kit (Illumina, America). Gene and transcript expression levels were quantified using RSEM software. GO and KEGG enrichment analyses were conducted using Goatools and Rstudio.

To validate the transcriptomic results, RT‐qPCR was performed using the LightCycler 480 (Roche, USA) system with TB Green Ex Taq Premix. The housekeeping genes gyrB and rpoD were used as internal controls. 13 differentially expressed genes (DEGs) were selected for verification. Primer sequences for both target and reference genes are listed in Table S3.

### 4.8 Molecular Docking and Dynamics Simulation

Molecular docking was performed using HADDOCK 2.4 [115], with the 70S E. *coli* ribosome (PDB ID: 7K00) [116] as the receptor and GF (GLFKIIKKIAKSF, predicted by AlphaFold [117]) as the ligand. Key functional regions of the ribosome, including the A‐site and PET, were selected for exploratory docking. Docking results were clustered to identify the lowest‐energy, structurally reasonable GF‐A‐site complex, which was used for subsequent simulations. Molecular dynamics simulations were carried out using GROMACS 2023.2 with the CHARMM36m force field [118]. To reduce computational cost, a 15 Å spherical region centered on GF was extracted, including all residues directly interacting with the peptide. The system was solvated with TIP3P water and 0.15 m K^+^/Cl^−^, then equilibrated under NVT and NPT ensembles at 310 K and 1 bar. Production simulations were run for 100 ns with weak positional restraints applied to peripheral residues, while GF and directly interacting residues remained fully flexible. Trajectories were analyzed for RMSD, hydrogen bonds, and interaction energies.

### 4.9 Cell‐Free Protein Synthesis Inhibition Assay

A cell‐free protein translation assay was performed using the PURExpress in vitro protein synthesis system (New England Biolabs, EU). Reaction mixtures were prepared according to the manufacturer's instructions and supplemented with DHFR DNA template. GF or an equal volume of nuclease‐free water was added to the reaction, respectively. Reaction products were collected and analyzed by SDS‐PAGE and Coomassie Brilliant Blue staining (Beyotime, China) to quantify DHFR expression. The extent of protein inhibition was used to assess the suppressive effect of GF on the protein translation process.

### 4.10 Synthesis of NH_2_‐MIL‐101

NH_2_‐MIL‐101 was synthesized following a previously reported procedure [77]. Briefly, 67.6 mg of FeCl_3_⋅6H_2_O and 45.3 mg of BDC‐NH_2_ were each dissolved in 10.0 mL of DMF. The two solutions were then mixed and sonicated for 15 min. The mixture was heated at 120 °C for 12 h. After the reaction, the reddish‐brown precipitate was collected by centrifugation at 8000 rpm for 5 min. The product was washed three times with DMF and ethanol to remove residual solvents and unreacted materials, then vacuum‐dried overnight at room temperature.

### 4.11 Synthesis and Loading Efficiency Determination of I@M, G@M, and IG@M

To prepare I@M, 2 mg of NH_2_‐MIL‐101 was dispersed in 5 mL of PBS (pH 7.4), followed by the addition of Irisin (8 µg/mL, 5 mL). The mixture was stirred at 4 °C overnight, and the resulting product was collected by centrifugation. For the synthesis of IG@M, the I@M precursor solution was prepared as described above. GF (160 µm) was then added, and the mixture was stirred for an additional 4 h at 4 °C. The final product was obtained by centrifugation, separating both the precipitate and the supernatant. To synthesize G@M, 2 mg of NH_2_‐MIL‐101 was dispersed in 10 mL of PBS, followed by the addition of GF (160 µm). The mixture was stirred for 4 h at 4 °C, and the product was collected by centrifugation. Irisin/FITC‐GF@M and FITC‐GF@M were prepared using the same protocols. All samples were lyophilized and stored at −20 °C for further use. The amount of unbound Irisin in the supernatants was measured using a human Irisin ELISA kit (Cusabio, Wuhan, China, CSB‐EQ027943HU) according to the manufacturer's protocol. Absorbance at 450 nm was recorded and converted to concentration using the standard calibration curve. Unbound FITC‐GF was determined by measuring fluorescence intensity with a microplate reader (TECAN, Spark), and concentrations were calculated based on a fluorescence–concentration calibration curve generated from FITC‐GF of known concentrations. All measurements were performed in triplicate.

The loading efficiency (LE%) of Irisin and GF was calculated by Equation 1:

(1) $$\mathrm{LE}\;\left(\%\right)=\frac{W_{initial}-W_{free}}{W_{initial}}\times 100\%$$

where *W_initial_* represents the total amount of Irisin or GF initially added, and *W_free_* represents the amount remaining in the supernatant after loading.

### 4.12 Characterization of M, I@M, G@M, and IG@M

The crystalline structure of NH_2_‐MIL‐101 was examined using XRD (D8 ADVANCE, Bruker, Germany). FTIR (Thermo Scientific, USA) was used to assess functional group characteristics. The morphology and microstructure of the particles were observed using SEM (TESCAN MAIA 3 GMU) and TEM (HT‐7800, Hitachi, Japan). Elemental composition was determined by EDS using a field emission SEM (Quanta FEG 250). Particle size distribution and Zeta potential were measured using a laser particle size analyzer (Mastersizer 2000, Malvern, UK).

### 4.13 Release Profile of Irisin and GF

To evaluate the release profiles of Irisin and GF, IG@M was suspended in PBS at different pH values (7.4 and 5.5). At predetermined time points from day 1 to day 7, 100 µL of supernatant was withdrawn and replaced with an equal volume of fresh PBS to maintain a constant volume. The release amounts of Irisin and FITC‐GF were quantified using the same methods applied for loading efficiency determination. The cumulative release efficiency was calculated using the following Equation 2:

(2) $$\mathrm{cumulative}\;\mathrm{release}\;\left(\%\right)=\frac{V_{total}\times C_{t}+V_{sample}\times \sum C_{i}}{W_{initial}}\times 100\%$$

where *V_total_* represents the total volume of PBS used as the release medium, *C_t_* represents the drug concentration in the supernatant at time t, *V_sample_* represents the volume collected at each time point, *C_i_* represents the concentration at each previous time point (i = 1, 2…, t−1), and *W_initial_* represents the initial amount of added Irisin or GF.

### 4.14 Protease Protection of Irisin and GF by IG@M

To evaluate the protective capacity of IG@M against proteolytic degradation, free Irisin, GF, and IG@M were separately incubated with trypsin for 1 h. Protein bands were visualized using SDS‐PAGE and Coomassie Brilliant Blue staining to assess the retention of Irisin in each group. AEBSF (4‐(2‐aminoethyl)benzenesulfonyl fluoride hydrochloride, Beyotime, China) was added to inactivate the residual trypsin, after which the treated GF and IG@M samples were incubated with S. *aureus* to determine whether the composite preserved the antibacterial activity of GF.

### 4.15 Biocompatibility Assessment

Log‐phase HOK, Cal27, and hPDLCs were seeded into 96‐well plates at a density of 2.5 × 10^4^ cells/mL. Each well received 200 µL of the cell suspension and was incubated for 24 h under standard culture conditions (37 °C, 5% CO_2_). After removing the medium, cells were treated with graded concentrations of M, I@M, G@M, or IG@M (each at 25 µg/mL). Following 48 h of incubation, cell viability was assessed using the CCK‐8 assay (Beyotime, China) according to the manufacturer's instructions. Absorbance was measured at 450 nm using a microplate reader.

Cell viability (%) was calculated by Equation 3:

(3) $$\mathrm{cell}\;\mathrm{viability}\;\left(\%\right)=\frac{A_{test}-A_{blank}}{A_{control}-A_{blank}}\times 100\%$$

where *A_test_* represents the absorbance of treated wells, *A_control_* represents the absorbance of untreated control wells, and *A_blank_* represents the background absorbance.

To assess apoptosis, cells were stained with the Annexin V/Caspase‐3 detection kit (Beyotime, China) following the manufacturer's protocol. Stained cells were imaged using an inverted fluorescence microscope (DMI3000 B, Leica, Germany).

### 4.16 Scratch Wound Healing Assay

hPDLCs were seeded into 6‐well plates at a density of 5 × 10^5^ cells/well. Once the cells reached full confluence, a straight scratch was made across the monolayer using a 100 µL pipette tip. After washing the wells three times with PBS to remove cell debris, the cells were cultured in serum‐free medium containing M, I@M, G@M, or IG@M. Images of the scratched area were captured under a microscope at 0, 24, and 48 h. The wound area was quantified using ImageJ software.

Cell migration efficiency was calculated using the Equation 4:

(4) $$\mathrm{relative}\;\mathrm{migration}\;\mathrm{rate}\;\%=\frac{S_{0}-S_{t}}{S_{0}}\times 100\%$$

where *S_0_* represents the wound area at 0 h and *S_t_* represents the wound area at the indicated time point.

### 4.17 Hemolysis Assay

1 mL suspension of 2% rabbit red blood cells (SenBeiJia Biological Technology Co., Ltd., Nanjing, China) was centrifuged and washed, then resuspended in 500 µL of saline. Equal volumes (500 µL) of red blood cell suspension and M, I@M, G@M, or IG@M solutions at various concentrations were mixed. Saline and deionized water served as the negative and positive controls, respectively. After incubation at 37 °C for 1 h, the samples were centrifuged at 3000 rpm for 10 min. The absorbance of the supernatant was measured at 540 nm using a microplate reader.

The hemolysis rate was calculated using the following Equation 5:

(5) $$\mathrm{hemolysis}\;\mathrm{ratio}\;\left(\%\right)=\frac{A_{test}-A_{control}}{A_{positive}-A_{control}}\times 100\%$$

where *A_test_* represents the absorbance of the test sample, *A_positive_* represents that of the positive control, and *A_control_* represents that of the negative control.

### 4.18 Cell Morphology Observation

hPDLCs were seeded in 6‐well plates at a density of 5 × 10^5^ cells/well. After 24 h of incubation the culture medium was replaced with medium containing M, I@M, G@M, or IG@M. Cells were further incubated for 24 h. Subsequently, the cytoskeleton was stained with Alexa Fluor 488 phalloidin, and nuclei were stained with DAPI. Cell morphology were observed using an inverted fluorescence microscope.

### 4.19 ROS Detection

The intracellular ROS levels were evaluated using a commercial ROS detection kit (Beyotime, China). After 24‐h treatment, cells were treated with 10 µm DCFH‐DA and incubated at 37 °C in the dark. A ROSUP‐treated group served as the positive control. After a 20‐min incubation, cells were thoroughly washed to remove excess probe. Fluorescent images were captured using an inverted fluorescence microscope.

### 4.20 Antibacterial Activity Assessment

Bacterial suspensions (1 × 10^6^ CFU/mL) were incubated with M, I@M, G@M, or IG@M at 37 °C for 24 h, with PBS‐treated samples serving as the negative control. After incubation, the suspensions were serially diluted tenfold and plated on corresponding agar media. After 24–48 h of incubation, colony‐forming units (CFUs) were enumerated. The antibacterial rate was calculated by Equation 6:

(6) $$\mathrm{antibacterial}\;\mathrm{rate}\;\left(\%\right)=\frac{N_{control}-N_{test}}{N_{control}}\times 100\%$$

where *N_control_* represents the number of bacterial colonies in the negative control group, and *N_test_* represents the number of colonies in the treatment group.

Each material group was co‐cultured with bacterial suspensions (1 × 10^6^ CFU/mL) in 96‐well plates for 48 h. Non‐adherent bacteria were removed by washing with PBS. The remaining biofilm was fixed and stained with 0.1% crystal violet, photographed, and then solubilized in 95% ethanol. Absorbance was measured at 595 nm to quantify biofilm biomass. The biofilm biomass rate was calculated by Equation 7:

(7) $$\mathrm{biofilm}\;\mathrm{biomass}\;\left(\%\right)=\frac{A_{test}-A_{blank}}{A_{control}-A_{blank}}\times 100\%$$

where *A_test_* represents the absorbance of the test groups, *A_control_* is the absorbance of the negative control group, and *A_blank_* is the absorbance of the blank control group.

To visualize mature biofilms, bacteria were first cultured for 24 h at 37 °C to allow biofilm formation. These pre‐formed biofilms were then treated with different materials for an additional 24 h. The samples were stained with the LIVE/DEAD BacLight Bacterial Viability Kit (Thermo Fisher Scientific, USA), and imaged in 3D using a CLSM (Zeiss LSM 780, Zeiss, Germany).

For morphological evaluation of bacteria within biofilms, samples co‐cultured with materials on coverslips were fixed in 2.5% glutaraldehyde. After dehydration through a series of ethanol concentrations (30%, 50%, 70%, 80%, 90%, and 100%), samples were sputter‐coated and examined under a SEM to assess bacterial membrane integrity and structural changes.

### 4.21 Bacterial Co‐Culture Model

THP‐1 cells were seeded into 6‐well plates at a density of 5 × 10^5^ cells per well and treated with 100 ng/mL phorbol 12‐myristate 13‐acetate (PMA) for 24 h to induce differentiation into macrophages. Based on assessments of cell viability and qPCR analysis of inflammatory cytokines, a multiplicity of infection (MOI) of 50 was selected as the optimal stimulation condition (Figure S9a–d). The macrophages were then co‐cultured with F. *nucleatum* for 2 h under antibiotic‐free conditions. After incubation, cells were thoroughly washed with PBS. Subsequently, the cells were treated with the respective materials (M, I@M, G@M, or IG@M) and incubated for an additional 24 h in culture medium supplemented with 10 µg/mL metronidazole. All samples were then used for downstream anti‐inflammatory and antioxidant analyses.

### 4.22 RNA Isolation and qPCR Analysis

Total RNA was isolated from each treatment group using an RNA extraction kit (Beyotime, Shanghai, China) following the provided guidelines. Reverse transcription was conducted using HiScript III RT SuperMix (Vazyme, China), and quantitative PCR was performed with Universal SYBR qPCR Master Mix (Vazyme, China). The relative expression of mRNAs was determined using the 2^−ΔΔCt method, with primer sequences provided in Table S4.

### 4.23 ELISA and Flow Cytometry

The culture supernatants were collected, and the levels of cytokines (TNF‐α, IL‐1β, and IL‐6) were measured using an Xmplex detection kit (XM‐Biotech, China). Cells were digested with Accutase and then centrifuged at 1000 rpm for 5 min. After resuspension, cells were treated with an Fc blocker at room temperature for 10 min, followed by staining with anti‐human CD86‐BV421 antibody (BD Biosciences, USA) at 4 °C in the dark for 30 min. Flow cytometric analysis was performed using a flow cytometer (MA900, SONY), and the proportion of M1‐polarized macrophages was quantified using FlowJo software.

### 4.24 Immunofluorescence Staining

Cells were fixed with 4% paraformaldehyde and blocked with goat serum at room temperature for 1 h. Samples were then incubated overnight at 4 °C with primary antibodies against iNOS (1:200, Proteintech), Nrf2 (1:200, Proteintech), RUNX2 (1:200, Abbkine), and OCN (1:200, MedChemExpress). After washing with PBS, cells were incubated in the dark at room temperature for 1 h with Alexa Fluor 594‐ or 488‐conjugated goat anti‐rabbit IgG secondary antibodies (1:200, Invitrogen, USA). Nuclei were counterstained and mounted using DAPI‐containing antifade mounting medium (Beyotime, China). Fluorescence images were captured using an upright metallurgical microscope.

### 4.25 ALP and Alizarin Red S Staining

hPDLCs (5 × 10^4^ cells per well) were seeded into 12‐well plates. Once the cells reached 70% confluence, the medium was replaced with osteogenic induction medium containing M, G@M, I@M, or IG@M. The osteogenic medium consisted of 10 nm dexamethasone, 10 mm β‐glycerophosphate, and 50 µg/mL ascorbic acid. Cells maintained in standard culture medium served as the negative control. The medium was refreshed every 3 days. On day 7, cells were rinsed with distilled water and fixed in 4% paraformaldehyde for 15 min. ALP staining was performed using a BCIP/NBT staining kit (Beyotime, China). In parallel, mineralization was evaluated on day 21 by repeating the washing and fixation steps, followed by ARS staining (Regenbio, China) to detect calcium nodule formation. Stained cells were examined and imaged using a light microscope. The culture plates were scanned using a flatbed scanner.

### 4.26 Western Blot

hPDLCs (5 × 10^5^ cells per well) were seeded into 6‐well plates and cultured under the same osteogenic induction conditions described above. On day 7, proteins were extracted from each group of cells. The samples were separated by SDS‐PAGE and transferred onto polyvinylidene fluoride (PVDF) membranes. After blocking, the membranes were incubated overnight at 4°C with primary antibodies against RUNX2 (1:1000, Abbkine), OSX (1:1000, Abbkine), OCN (1:1000, MedChemExpress), COL1 (1:2000, Proteintech), and GAPDH (1:10 000, Abbkine). After incubation with HRP‐conjugated secondary antibodies, protein bands were visualized via an enhanced chemiluminescence (ECL) detection system.

### 4.27 Establishment of Mouse Periodontitis Model

The ligature‐induced murine periodontitis model was employed to evaluate inflammatory responses and alveolar bone changes, as disease progression is primarily driven by bacterial accumulation at the ligature site [119]. All animal procedures were approved by the Institutional Animal Care and Use Committee (IACUC) of Nanjing Medical University (Approval No. IACUC‐2503025) and conducted in accordance with ethical guidelines. 20 male C57BL/6 mice were randomly assigned to four groups: healthy control, periodontitis, F. *nucleatum* infection, and IG@M treatment. Under anesthesia with 1% pentobarbital sodium, periodontitis was induced by ligating the cervical region of the maxillary second molars using 5‐0 surgical sutures. For F. *nucleatum* infection and IG@M treatment groups, subgingival injections of F. *nucleatum* suspension (10^7^ CFU/mL, 10 µL) were administered every other day for a total of three doses during the ligation period. The ligatures were removed 1 week after placement. Mice in the IG@M treatment group received local drug administration following ligature removal. 10 µL of IG@M suspension was injected into six sites surrounding the periodontal pocket using a 25 µL microsyringe (Hamilton, USA). Other groups received equal volumes of sterile saline. Injections were performed every 4 days, for a total of seven administrations. After 4 weeks of treatment, all mice were euthanized with an overdose of anesthesia. Maxillary bone samples and major organs were collected (Figure S7a). All tissues were fixed in 4% paraformaldehyde for 24 h and processed for subsequent analyses.

### 4.28 Micro‐CT Analysis

Micro‐CT scans were performed on the ligated molars and surrounding maxillary alveolar bone. 3D reconstruction of the scanned images was conducted using Materialise Mimics (version 21.0). Quantitative 2D analysis was carried out with CTAn to valuate alveolar bone resorption. Parameters evaluated included BV/TV, BMD, Tb.N, and the vertical distance from the CEJ to the ABC.

### 4.29 Histological and Immunohistochemical Analysis

After micro‐CT scanning, tissue samples were decalcified, dehydrated, embedded and sectioned according to standard protocols. Histopathological evaluation of the heart, liver, spleen, lungs, and kidneys was performed to assess the biocompatibility of the IG@M. H&E staining and Masson trichrome staining were used to examine histological changes and collagen deposition. Giemsa staining was performed to visualize bacterial infiltration within the tissues. Immunohistochemical staining was conducted to detect the expression of iNOS, TNF‐α, COL1, and OCN in periodontal tissues. Quantitative analysis of staining intensity was performed using ImageJ software.

### 4.30 Statistical Analysis

Data were inspected for normality and homogeneity of variance prior to analysis. No data transformation was applied unless otherwise specified. All data are presented as mean ± SD. The sample size for each experiment is provided in the corresponding figure legends. Statistical analyses were performed using one‐way ANOVA or unpaired t‐tests when appropriate. For multiple group comparisons, Dunnett's or Tukey's post hoc tests were used to assess significant differences between groups. All graphs and statistical outputs were generated using GraphPad Prism 10.0 (GraphPad Software, USA) and OriginPro 2021 (OriginLab Corp., USA).

## Funding

This work was supported by the Project of Jiangsu Provincial Commission of Health (No. M2022060), the National Natural Science Foundation of China (No. 81801029), the Anhui Province Key Cultivation Project for Excellent Young Teachers in Universities (No. YQZD2023060), Jiangsu Province Capability Improvement Project through Science, Technology and Education‐Jiangsu Provincial Research Hospital Cultivation Unit (YJXYYJSDW4) and Jiangsu Provincial Medical Innovation Center (CXZX202227).

## Conflicts of Interest

The authors declare no conflict of interest.

## Supporting information




**Supporting File**: advs73997‐sup‐0001‐SuppMat.docx.

## Data Availability

The data that support the findings of this study are available from the corresponding author upon reasonable request.
