# Supplementary material for: Cascade Therapy of Periodontitis via Sequential Release of Ribosome‐Targeting Antimicrobial Peptide and Irisin From a Multifunctional MOF‐Based System
Source: Adv Sci (Weinh). 2026 Jan 27;13(18):e21553. doi: 10.1002/advs.202521553 (PMC13042883; doi:10.1002/advs.202521553)
Supplement: Supplementary file 1 — Supporting File: advs73997‐sup‐0001‐SuppMat.docx. [file ADVS-13-e21553-s001.docx]

Supporting Information

Cascade Therapy of Periodontitis via Sequential Release of Ribosome-targeting Antimicrobial Peptide and Irisin from a Multifunctional MOF-based System

*Yan Chen^†^, Zheng Xu^†^, Yunmo Xue, Shanshan Liu, Xiang Zhang, Jingyao Guo, Minhui Yao, Yue Liu, Xiaolin Lu, Jieshu Qian, Qian Ma^*^*


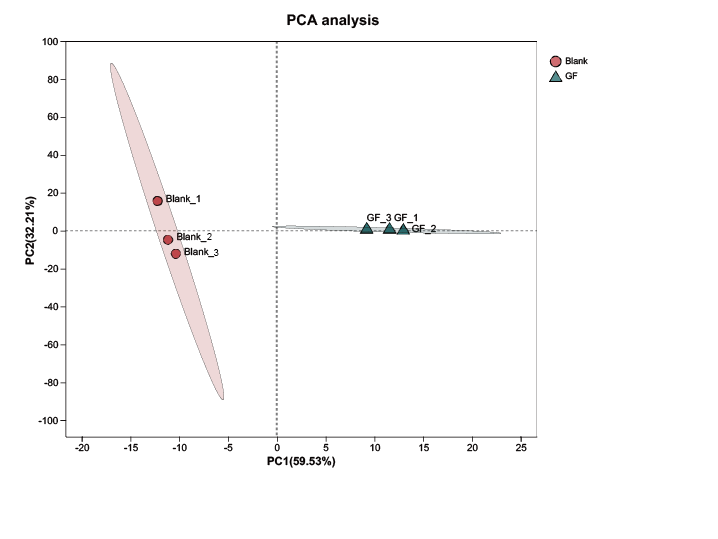


Figure S1. PCA of transcriptomes from the GF-treated and control groups.


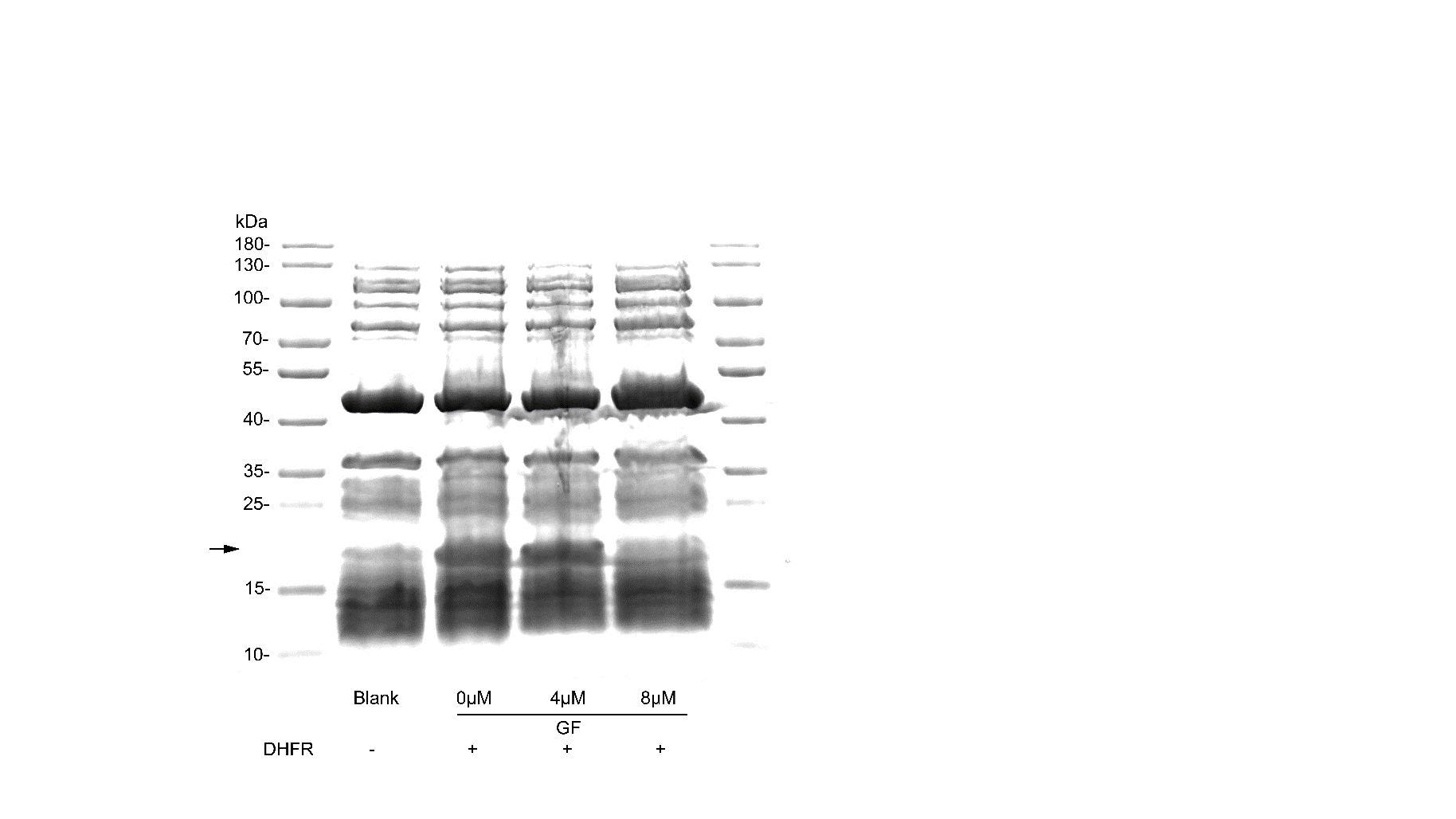


Figure S2. GF inhibits ribosomal translation in a dose-dependent manner.


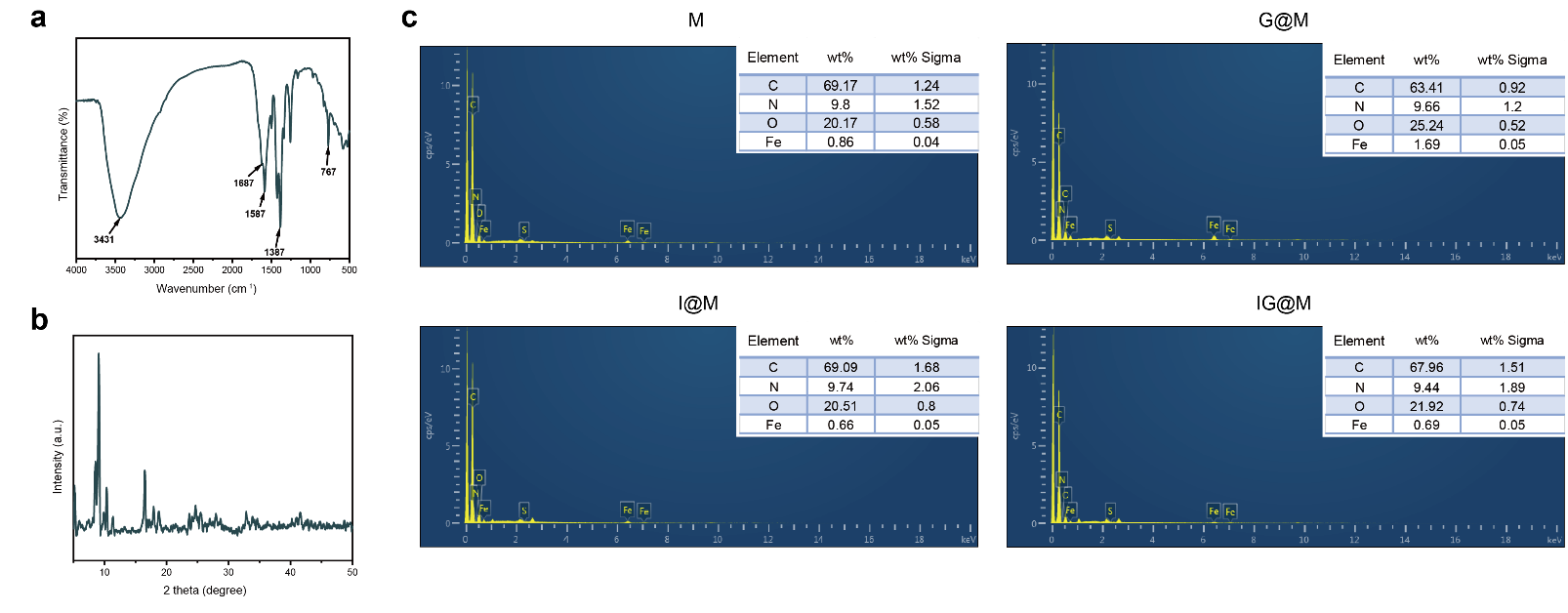


Figure S3. a) FTIR spectrum of NH_2_-MIL-101. b) XRD pattern of NH_2_-MIL-101. c) EDS mapping of M, G@M, I@M, and IG@M.


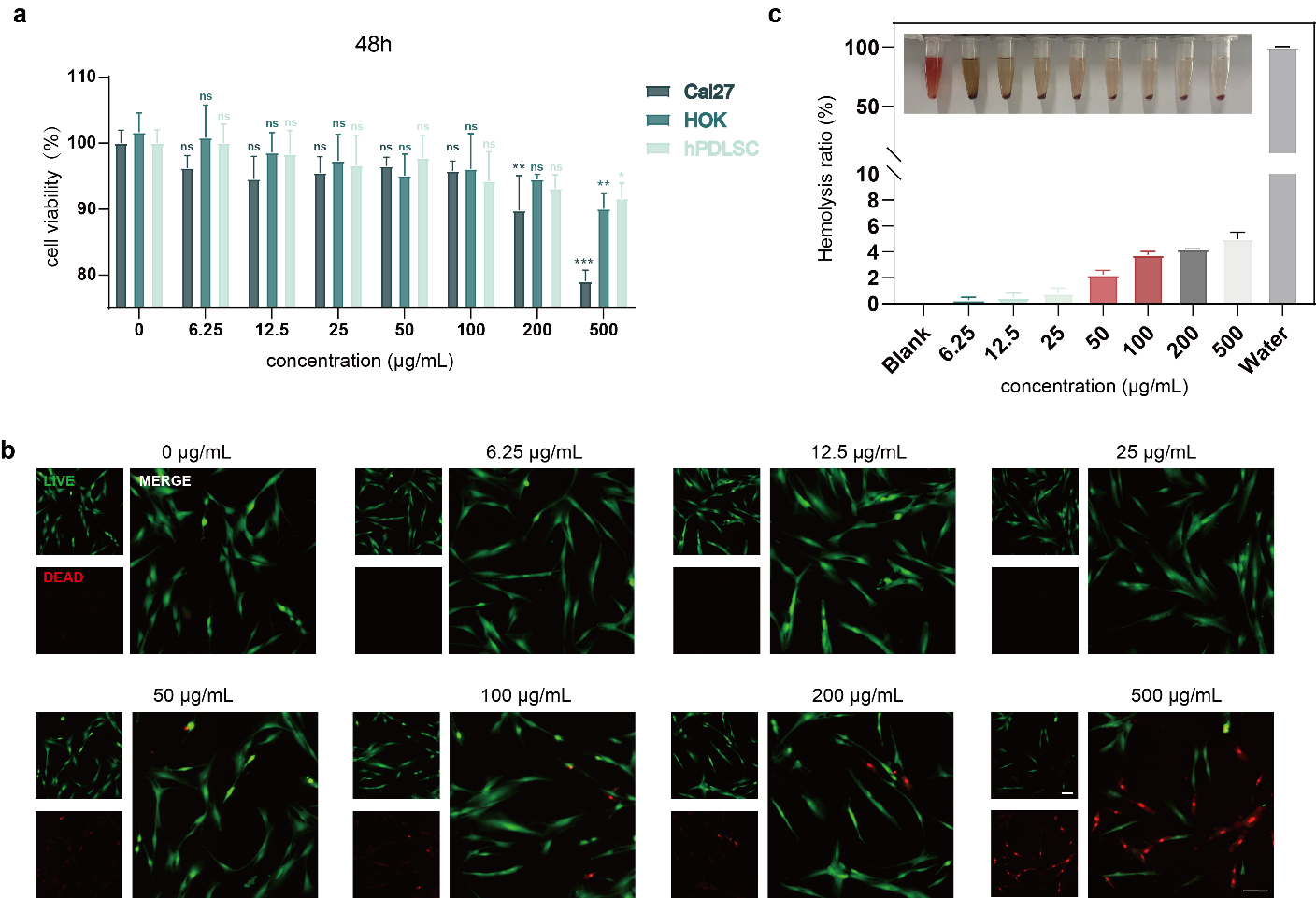


Figure S4. a) CCK-8 analysis of NH_2_-MIL-101 at 48 h. b) Live/dead fluorescent staining images at 24h (scale bar: 100 µm). c) Hemolysis test. Data are presented as means ± SD (n = 3). ns p > 0.05, *p < 0.05, **p < 0.01, ***p < 0.001.


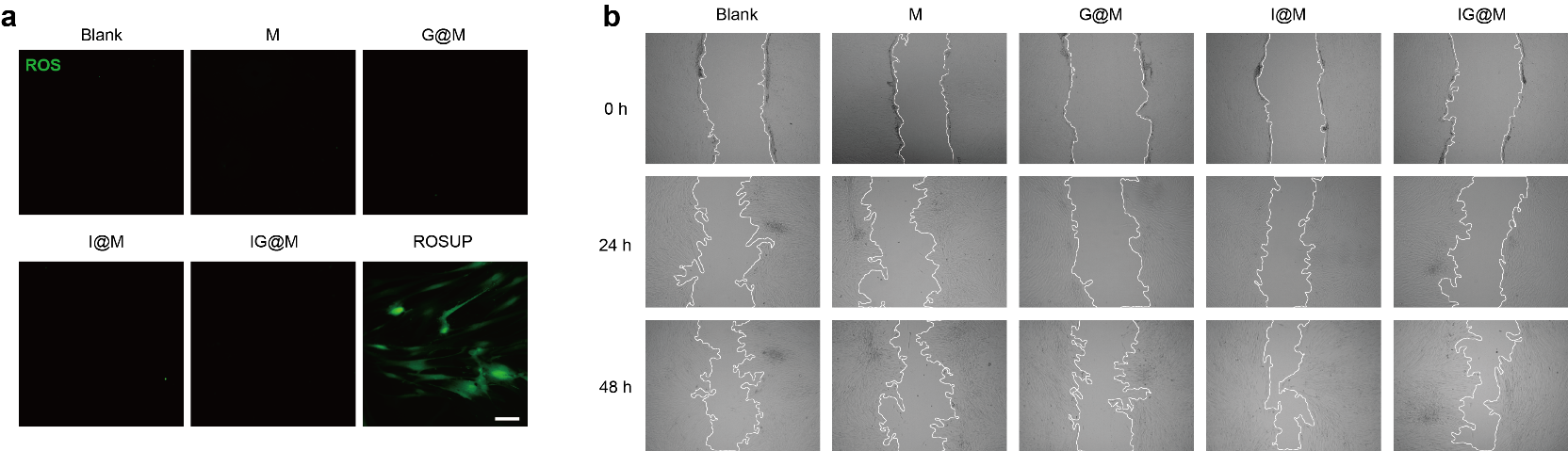


Figure S5. a) ROS levels in M, G@M, I@M, and IG@M treatment groups (scale bar: 100 µm). b) Representative images from cell migration assays.


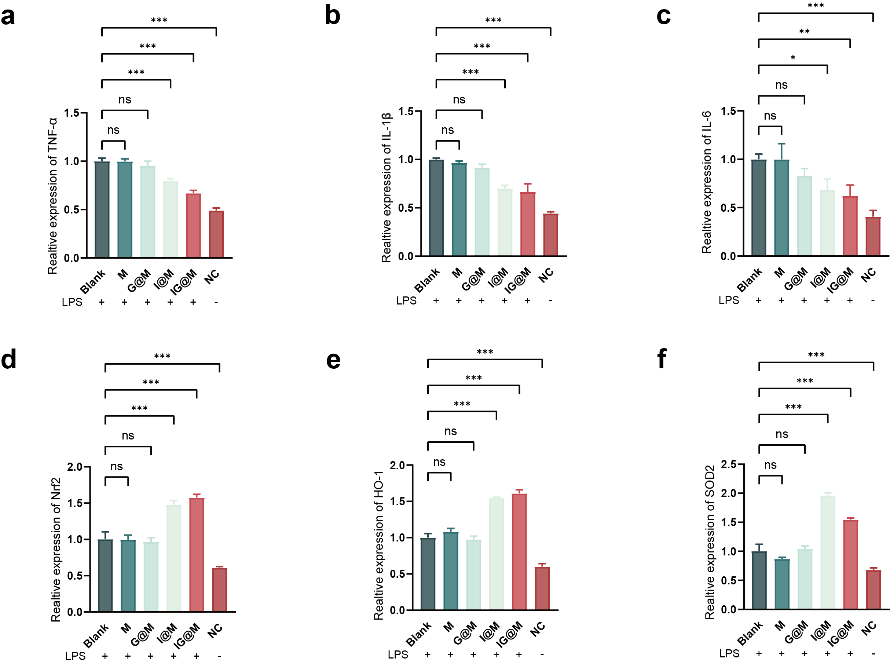


**Figure** S6. a-f) qPCR analysis of TNF-α, IL-1β, IL-6, Nrf2, HO-1, and SOD2 expression in THP-1 cells after LPS stimulation. Data are presented as means ± SD (n = 3). Statistical significance: ns, *p* > 0.05; **p* < 0.05; ***p* < 0.01; ****p* < 0.001.


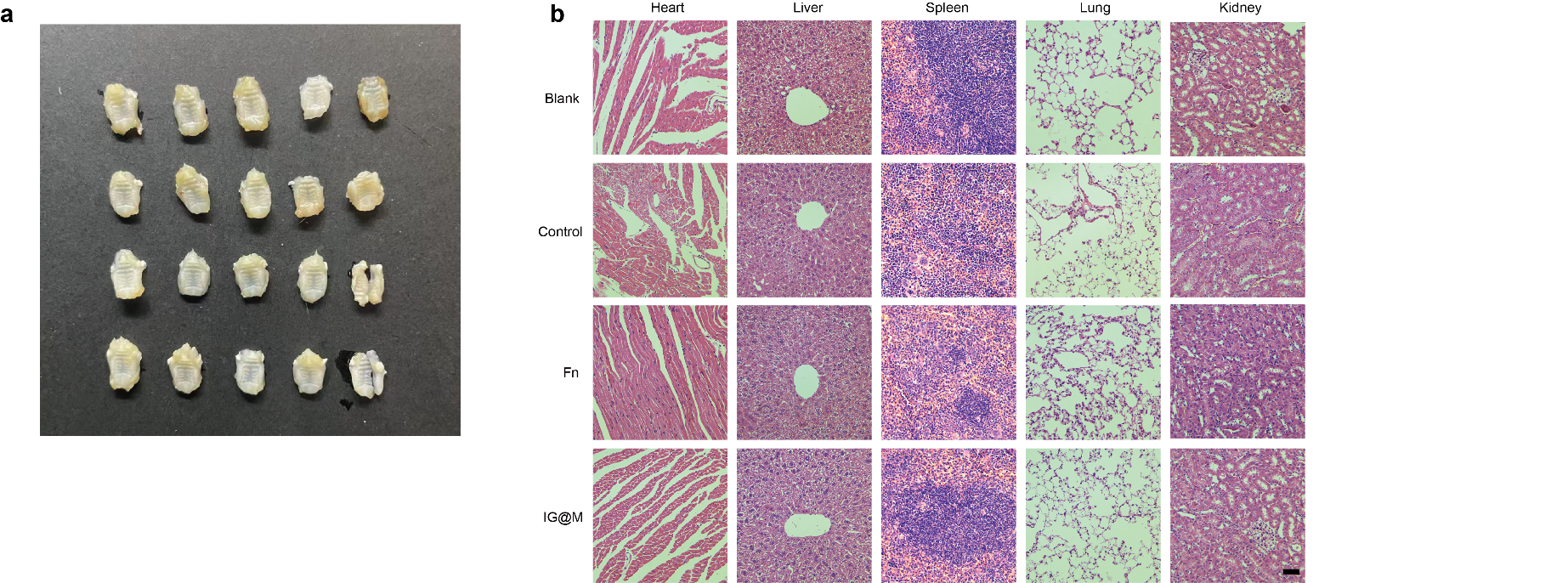


Figure S7. a) Image of maxillary bone samples. b) H&E staining of the heart, liver, spleen, lung, and kidney (scale bar: 50 µm).


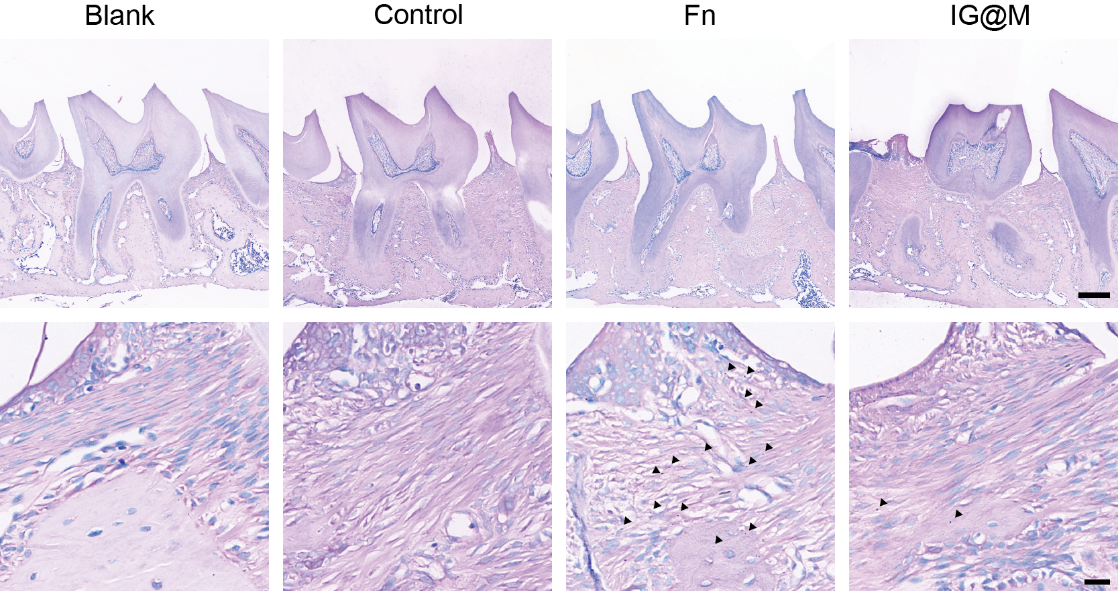


Figure S8. Giemsa staining showing bacterial infiltration in tissue sections. Black arrows indicate bacteria. (scale bars: 200 µm for main images; 20 µm for magnified views)


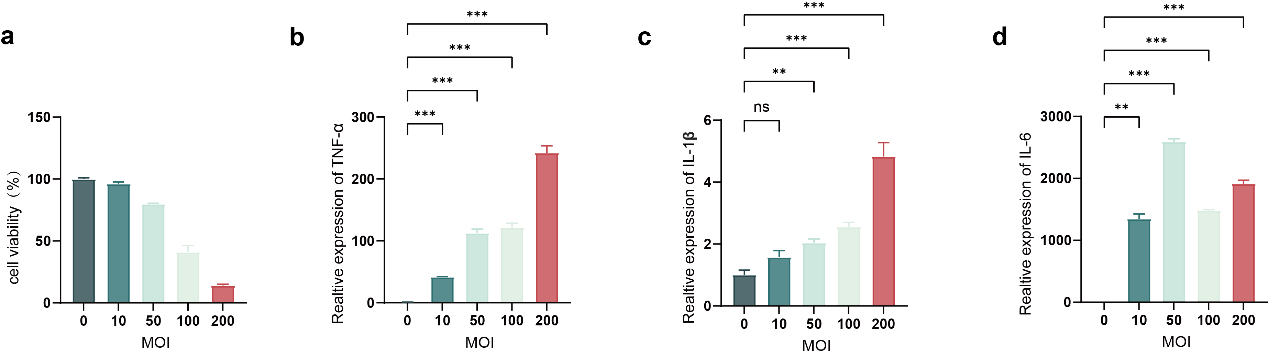


Figure S9. Cell viability and gene expression of THP-1 cells under different MOI. a) CCK-8 assay of cell viability. b-d) qPCR analysis of TNF-α, IL-1β, and IL-6 expression. ns, *p* > 0.05; ***p* < 0.01; ****p* < 0.001 (n = 3).

Table S1. GO enrichment analysis of DEGs from the transcriptome.

| Gene ID | Gene name | Gene description | Log2FC  (GF/Blank) | Padjust | Regulate |
| --- | --- | --- | --- | --- | --- |
| C7Y58_RS00655 | rpsE | 30S ribosomal protein S5 | -5.690077348 | 1.85E-11 | down |
| C7Y58_RS00660 | rplR | 50S ribosomal protein L18 | -5.804850954 | 3.66E-09 | down |
| C7Y58_RS00675 | rpsN | 30S ribosomal protein S14 | -5.939079581 | 7.49E-08 | down |
| C7Y58_RS00665 | rplF | 50S ribosomal protein L6 | -5.966339916 | 7.49E-08 | down |
| C7Y58_RS00685 | rplX | 50S ribosomal protein L24 | -6.468332833 | 2.15E-07 | down |
| C7Y58_RS00705 | rplP | 50S ribosomal protein L16 | -5.643260588 | 2.60E-07 | down |
| C7Y58_RS00680 | rplE | 50S ribosomal protein L5 | -6.41482137 | 3.79E-07 | down |
| C7Y58_RS00690 | rplN | 50S ribosomal protein L14 | -5.349986828 | 4.31E-07 | down |
| C7Y58_RS04385 | C7Y58_RS04385 | adhesion protein FadA | -1.059341011 | 0.150244723 | down |
| C7Y58_RS01520 | C7Y58_RS01520 | DMT family transporter | 2.117802052 | 2.78E-05 | up |
| C7Y58_RS04030 | C7Y58_RS04030 | sensor histidine kinase | 1.97069567 | 6.54E-05 | up |
| C7Y58_RS08875 | C7Y58_RS08875 | L-lactate dehydrogenase | 1.905886585 | 8.31E-06 | up |
| C7Y58_RS10200 | pfkB | 1-phosphofructokinase | 3.14447345 | 3.08E-05 | up |

Table S2. Comparison of HADDOCK docking results for GF at the PET site and A-site before and after optimization.

|  | HADDOCK score | RMSD | Vdw energy | Electrostatic energy | Desolvation energy | Restraint’s violation energy | Buried Surface Area | Z-Score |
| --- | --- | --- | --- | --- | --- | --- | --- | --- |
| GF-PET | -59.2 ± 11.5 | 28.6 ± 0.0 | -33.8 ± 6.2 | -189.9 ± 36.5 | 2.9 ± 0.5 | 97.1 ± 36.9 | 1025.7 ± 118.8 | -0.4 |
| GF-A site (before optimization) | -76.7 ± 2.6 | 0.6 ± 0.4 | -50.0 ± 1.9 | -310.5 ± 25.9 | 3.3 ± 2.7 | 10.5 ± 7.5 | 1226.2 ± 57.3 | -1.6 |
| GF-A site (after optimization) | -76.9 ± 2.4 | 1.1 ± 0.1 | -49.2 ± 1.6 | -318.1 ± 18.9 | 4.1 ± 1.4 | 0.2 ± 0.1 | 1280.4 ± 36.9 | -1.5 |

Table S3. Primer sequences of target and reference genes in F. *nucleatum*.

| Gene | 5‘-3’ | 3‘-5’ |
| --- | --- | --- |
| rpoD | ACTCCTGTTGGAAGCGAAGA | AGCTTCATAAGGGCTTGTTGT |
| gyrB | GGGGGATTACATGGAGTTGGA | TCAAGCCACTCAGAAAGGGC |
| rpsN | GTCAATTAGATGGTAGACCAAGAGG | AGCGCCAGCAAGTTGTCTAA |
| rpsQ | GAAAAGTGAGAGAAGGAATAGTTGT | AGCTACATTTTCTTCATCATGAGC |
| rpmD | AACTGTAAAGTCGCTAGGGC | ATGCTTGCACCTCCTCAACT |
| rplC | GGCTGGACAACATGGAAATGC | GCTCCTGGAACTGCTCCTTTT |
| rpsC | GCAGTAGCAACAGCTCATACAA | GCTTCCCCTCCTTCTTTCTT |
| rplV | TGCCAAGAGCAATGGGAAGA | TGCCACTGTGATATGAGCTGTT |
| rplF | TCATGGAACAACAAGAGCCT | TGCTCTGTACCCAACCCCTA |
| rplP | CCCTGACAAACCAATCACAGC | CCCAACCTTCAACGTTTCCTT |
| rplE | AGATGCTGCTATGGCTGATT | TGCTCCAATAGGCATTCCTTC |
| C7Y58_RS01520 | TGGTGGTAATTGGTGGGCAA | AGAAAACAAAGGCAACCCCT |
| C7Y58_RS04030 | TGATGGAATGGACGATTGGGT | AGATACTCATGAAGTGCTTGGT |
| C7Y58_RS08875 | TCTATACAGGAGTTCCAGCCAT | GCACAAGCATCTTCAAAGCC |
| pfkB | TGTTGGTGCAGGAGATTCAGT | GTTCCACAAGCCACTGCAAA |

Table S4. Primer sequences of target and reference genes in cells.

| Gene | 5‘-3’ | 3‘-5’ |
| --- | --- | --- |
| GAPDH | AAGCCTGCCGGTGACTAAC | GCGCCCAATACGACCAAATC |
| TNF-α | CCTCTCTCTAATCAGCCCTCTG | GAGGACCTGGGAGTAGATGAG |
| IL-1β | ATGATGGCTTATTACAGTGGCAA | GTCGGAGATTCGTAGCTGGA |
| IL-6 | GTACATCCTCGACGGCATCTCA | GCACAGCTCTGGCTTGTTCCTC |
| iNOS | CGCATGACCTTGGTGTTTGG | CATAGACCTTGGGCTTGCCA |
| Nrf2 | AGGTTGCCCACATTCCCAAA | AACGTAGCCGAAGAAACCTCA |
| HO-1 | GCTCAACATCCAGCTCTTTGA | CAACTGTCGCCACCAGAAAG |
| SOD2 | AAACCTCAGCCCTAACGGTG | GCTTCCAGCAACTCCCCTTT |
| NQO1 | CCCTGCAGTGGTTTGGAGT | CACTGCCTTCTTACTCCGGAAGG |
| ALP | AACATCAGGGACATTGACGTG | GTATCTCGGTTTGAAGCTCTTCC |
| COL1 | GAGGGCCAAGACGAAGACATC | CAGATCACGTCATCGCACAAC |
| BMP2 | ACCCGCTGTCTTCTAGCGT | TTTCAGGCCGAACATGCTGAG |
| OSX | CCTCTGCGGGACTCAACAAC | AGCCCATTAGTGCTTGTAAAGG |
| RUNX2 | TGGTTACTGTCATGGCGGGTA | TCTCAGATCGTTGAACCTTGCTA |
